# Genome-Based Metabolic Reconstruction of a Novel Uncultivated Freshwater Magnetotactic coccus “*Ca*. Magnetaquicoccus inordinatus” UR-1, and Proposal of a Candidate Family “*Ca*. Magnetaquicoccaceae”

**DOI:** 10.3389/fmicb.2019.02290

**Published:** 2019-10-02

**Authors:** Veronika Koziaeva, Marina Dziuba, Pedro Leão, Maria Uzun, Maria Krutkina, Denis Grouzdev

**Affiliations:** ^1^Research Center of Biotechnology of the Russian Academy of Sciences, Institute of Bioengineering, Moscow, Russia; ^2^Department of Microbiology, University of Bayreuth, Bayreuth, Germany; ^3^Instituto de Microbiologia Paulo de Góes, Universidade Federal Do Rio de Janeiro, Rio de Janeiro, Brazil; ^4^Faculty of Biology, Lomonosov Moscow State University, Moscow, Russia

**Keywords:** magnetotactic bacteria (MTB), magnetosome, Candidatus Etaproteobacteria, Candidatus Magnetaquicoccaceae, magnetotactic cocci, *Magnetococcales*, magnetosome genomic island, AAI classifications

## Abstract

Magnetotactic bacteria are widely represented microorganisms that have the ability to synthesize magnetosomes. The magnetotactic cocci of the order *Magnetococcales* are the most frequently identified, but their classification remains unclear due to the low number of cultivated representatives. This paper reports the analysis of an uncultivated magnetotactic coccus UR-1 collected from the Uda River (in eastern Siberia). Genome analyses of this bacterium and comparison to the available *Magnetococcales* genomes identified a novel species called “*Ca*. Magnetaquicoccus inordinatus,” and a delineated candidate family “*Ca*. Magnetaquicoccaceae” within the order *Magnetococcales* is proposed. We used average amino acid identity values <55–56% and <64–65% as thresholds for the separation of families and genera, respectively, within the order *Magnetococcales*. Analyses of the genome sequence of UR-1 revealed a potential ability for a chemolithoautotrophic lifestyle, with the oxidation of a reduced sulfur compound and carbon assimilation by rTCA. A nearly complete magnetosome genome island, containing a set of *mam* and *mms* genes, was also identified. Further comparative analyses of the magnetosome genes showed vertical inheritance as well as horizontal gene transfer as the evolutionary drivers of magnetosome biomineralization genes in strains of the order *Magnetococcales*.

## Introduction

Magnetotactic cocci are one type of magnetotactic bacteria (MTB). Since the first description of MTB (Blakemore, 1975), many studies have shown that magnetotactic cocci are the most frequent morphotype among other MTB, both in freshwater (Moench and Konetzka, 1978; Spring et al., 1995; Flies et al., 2005a,b; Lin and Pan, 2009, 2010) and marine habitats (Spring et al., 1998; Abreu et al., 2012; Zhang et al., 2012; Du et al., 2017). However, only five representatives—*Magnetococcus marinus* MC-1^T^ (Frankel et al., 1997), *Magnetofaba australis* IT-1 (Morillo et al., 2014), “*Ca*. Magnetococcus massalia” MO-1 (Lefèvre et al., 2009) and strains PR-3 and SS-1 (Lefèvre et al., 2014) have been isolated in axenic cultures from hypersaline lagoon and marine habitats. Due to the difficulty of isolating MTB in an axenic culture, and especially the freshwater cocci forms, these studies have mostly been carried out using molecular methods for the analysis of environmental samples. However, magnetotactic cocci have a high swimming ability, so they can be easily isolated from non-magnetotactic bacteria using magnetic separation (Lin et al., 2008).

Early studies of the phylogeny of magnetotactic cocci described them as a separate clade within the class *Alphaproteobacteria* (Amann et al., 2006), but genome sequencing and analysis of three cultivated cocci led to a proposal to classify them as a separate class (“*Candidatus* Etaproteobacteria”) within the *Proteobacteria* phylum due to the mosaic origin of their genomes (Ji et al., 2017). Recently, 12 metagenome assembled genomes (MAGs) have been obtained for freshwater strains. The analysis confirmed that the order *Magnetococcales* belongs to the novel “*Ca*. Etaproteobacteria” class, rather than to the class *Alphaproteobacteria* (Lin et al., 2018).

MTB of the order *Magnetococcales* generally have an ovoid shape and usually synthesize magnetosomes with elongated prismatic and elongated octahedral crystal morphology (Pósfai et al., 2013); however, strain SHHC-1 demonstrates a large variety of magnetosome shapes within the same cell (Zhang et al., 2017). Rod-shaped bacteria potentially belonging to the order *Magnetococcales* have also been described (Zhang et al., 2013). The magnetosome arrangement within the cells varies from strain to strain: they are organized in one, two, or more chains and can be assembled as a dispersed cluster (Pan et al., 2008; Lefèvre et al., 2009; Lin and Pan, 2009; Abreu et al., 2012; Zhang et al., 2012, 2017; Morillo et al., 2014; Du et al., 2017; Kozyaeva et al., 2017). Many magnetotactic cocci have sulfur globules, indicating their ability to use reduced sulfur compounds, and phosphorus-rich inclusions that sometimes occupy most of the cell volume (Cox et al., 2002; Dziuba et al., 2013; Zhang et al., 2017). Cultivated magnetotactic cocci are microaerophiles and use thiosulfate for autotrophic growth, while using acetate and, in the case of IT-1, succinate for heterotrophic growth (Williams et al., 2006; Lefèvre et al., 2009; Morillo et al., 2014). The ability to accumulate a large amount of phosphorus indicates the important role of magnetotactic cocci in the phosphorus cycle. In particular, they can function as a “bacterial shuttle,” moving phosphorus from the water surface to deeper anaerobic layers (Rivas-Lamelo et al., 2017; Schulz-Vogt et al., 2019).

Magnetotactic cocci are diverse in their phylogeny as well as in their morphology. Attempts have been made to associate the 16S rRNA sequence with the ultrastructure of cells and crystallographic properties of their magnetosomes using fluorescent *in situ* hybridization (FISH) and other methods of FISH coupled with transmission and scanning electron microscopy (FISH-TEM and FISH-SEM). These approaches have been successfully used to identify cultured and uncultured MTB (Spring et al., 1998; Woehl et al., 2014; Li J. et al., 2017; Zhang et al., 2017; Li et al., 2019), and marine cocci have had their phylogenetic classification associated with their cell morphology and detailed characterization of magnetosome crystals (Abreu et al., 2012; Zhang et al., 2012, 2017). However, no clear results have yet been achieved for freshwater magnetotactic cocci (Spring et al., 1995; Lin and Pan, 2009). The number of genomic sequences associated with magnetotactic cocci has significantly increased, but information remains scarce regarding the cell and magnetosome structure of these MTB (Lin et al., 2018).

This paper reports the culture-independent characterization of an uncultivated magnetotactic coccus isolated from sediments of the Uda River (Eastern Siberia, Russia). FISH-TEM analysis associated its 16S rRNA gene sequence and the phenotype while reconstruction and analysis of the genome of this novel magnetotactic bacterium provided the first link between the genomic data of an uncultivated freshwater magnetotactic coccus with a specific phenotype. In particular, this genome analysis enabled the study of magnetosome biosynthesis genes in this organism in connection with the magnetosome organization within the cells, as revealed by TEM, thereby providing novel data to supplement previously published research on this group of MTB.

A comparison of the genome of the novel magnetotactic coccus with genomes of similar MTB available in public databases revealed several phylogenetic subgroups within the class *Magnetococcales* at the family level. Using the guidelines for the taxonomic description of uncultivated microbes provided by Konstantinidis et al. (2017), a candidate species for the novel magnetotactic coccus “*Ca*. Magnetaquicoccus inordinatus” UR-1 is described, and the genus “*Ca*. Magnetaquicoccus” and the family “*Ca*. Magnetaquicoccaceae” are proposed.

## Materials and Methods

### Sample Collection and DNA Extraction

MTB-containing sediment was collected in August 2012 from the Uda River in the city of Ulan-Ude, Eastern Siberia (51.8229 °N, 107.6199 °E). Physicochemical parameters of the water in the sampling site were provided by the Hydrochemical Institute (Rostov-on-Don, Russia). Surface sediments were collected near the shore from a water depth of ~0.5 meters and transferred to a 3-liter glass jar at a sediment: water ratio of ~1:3. The jars were stored in the laboratory at room temperature (~25°C) in dim light for 3 months. The enriched fraction of MTB cells was obtained by placing the south pole of the magnet on the outside of the jar, at the sediment-water interface. After 1 h, the spot of magnetotactic bacteria was collected using a Pasteur pipette. Magnetotactic properties were evaluated by observing the changes in bacterial motion in response to rotation of a magnet located on the stage of an Eclipse E200 light microscope (Nikon, Japan). A fraction of the collected cells (about 10 μl) were used for TEM; some cells were fixed in 3% paraformaldehyde for 1.5 h for coordinated FISH-TEM, and a third fraction was purified from non-magnetotactic microorganisms using the “race track” technique (Wolfe et al., 1987). The purified cells were used to isolate genomic DNA using a modified Birnboim-Doly alkaline method with a Wizard technique (Promega, USA) (Boulygina et al., 2002). Purified DNA was stored at −20°C.

### Clonal Library Construction and Phylogenetic Analysis of 16S rRNA Gene Sequences

The 16S rRNA gene sequences were amplified using universal primers 27F (5'-AGAGTTTGATCCTGGCTCAG-3') and 1492R (5'-AAGGAGGTGATCCAGCCGCA-3') (Lane, 1991). The obtained PCR products were purified with a Wizard PCR Prep kit (Promega, USA). Purified PCR products were ligated into a pGEM-T Easy System vector (Promega, USA) and cloned into *Escherichia coli* strain DH10B cells. Sequencing was performed on an ABI3730 DNA Analyzer sequencer (Applied Biosystems, USA) using the BigDye Terminator v3.1 Cycle Sequencing Kit (Applied Biosystems, USA) and the universal primers M13F, M13R, 530F, and 519R (Sambrook et al., 1989). The presence of chimeric sequences was verified using the Bellerophon online service (Huber et al., 2004). The obtained sequences were grouped in operational taxonomic units (OTUs) using identity threshold 99%. Obtained OTUs were aligned with 16S rRNA gene sequences of *Magnetococcales* strains using MAFFT (Katoh and Standley, 2013), and the maximum-likelihood tree was inferred using the GTR+F+I+G4 model recommended using ModelFinder (Wong et al., 2017) in IQ-Tree (Nguyen et al., 2015). Branch supports were obtained with 10,000 ultrafast bootstraps (Hoang et al., 2017). The 16S rRNA gene sequences of the OTUs were deposited in the GenBank database under accession numbers MK813936 and MK813937.

### Phylogenetic and Morphological Correlation of UR-1 Cells

To determine which cell the 16S rRNA sequence named UR-1 came from, a FISH-TEM analysis was performed on the same sample. Approximately 10 μl of fixed magnetically concentrated cells were added to a center-marked copper grid previously covered with Formvar film. After air drying for 5 min, the residual sample not attached to the grid was removed with filter paper, and a thin layer of sputtered carbon was placed on top of the sample (Balzers CED-030, Liechtenstein). The grids were store at room temperature in a vacuum chamber for 2 weeks before being used for FISH. The hybridization reaction was performed using the conditions and buffers described by Pernthaler et al. (2001) using a formamide concentration of 30% in the hybridization buffer, and a probe final concentration of 0.2 μg/ml (Pernthaler et al., 2001). After washing with washing buffer, the sample was stained with 4,6-diamidino-2-phenylindole (DAPI) at a final concentration of 0.1 μg/ml for 5 min and carefully washed with deionized water. For UR-1 cells identification, a specific probe, Uda54-3 (5' Cy3-CAAGAGCAATTCCAGGGTTAAGCCCTGGGCTT-3'), was designed and used as template to retrieve the 16S rRNA sequence MK813936 from the sequence. The positive control for the hybridization reaction was a mixture containing the bacterial universal probes EUB388I, EUB388II, and EUB388III (Daims et al., 1999) labeled with Alexa 488. The negative control was the same hybridization reaction using the Uda54-3 probe and EUB probes in a sample containing only *Escherichia coli* cells (Supplementary Figure S1). After performing the FISH reaction, the grid was placed between a glass slide and a coverslip and images were obtained using an AxioImager microscope (Zeiss, Germany) equipped with an AxioCam Mrc (Zeiss, Germany). The same grids used to perform FISH were placed on a Morgagni transmission electron microscope (FEI, USA) operated at 80 kV and images were obtained using a MegaView G2 CCD camera (Olympus, Japan) from the same area where the FISH had already been observed.

### Transmission Electron Microscopy

For conventional TEM, magnetically enriched cells were added to a Formvar-coated copper grid and imaged on a JEM-100CX (JEOL, Japan) transmission electron microscope operated at 80 kV. the TEM images were processed using ImageJ software to determine cell and magnetosome lengths, widths, and shape factors (width/length). Grids for high-resolution (HR) TEM were prepared as described for conventional TEM, and HR images were acquired using a Tecnai G2 F20 FEG transmission electron microscope (FEI, USA) operated at 200 kV and equipped with a 4k × 4k Gatan UltraScan 1,000 CCD camera. The HR images were analyzed using the Digital Micrograph software (Gatan, USA).

### Genome Sequencing, Assembly, and Annotation

DNA libraries were constructed with the NEBNext DNA library prep reagent set for Illumina, following the kit protocol. Sequencing was undertaken using the Illumina HiSeq 1500 platform with pair-end 150-bp reads. Raw reads were quality checked with FastQC v. 0.11.7 (http://www.bioinformatics.babraham.ac.uk/projects/fastqc/), and low-quality reads were trimmed using Trimmomatic v. 0.36 (Bolger et al., 2014). The quality-filtered reads were assembled *de novo* with metaSPAdes v. 3.12.0 using the default settings (Nurk et al., 2017). Genome statistics were evaluated using an automatic assembly quality evaluation tool (QUAST) (Gurevich et al., 2013). The assembled metagenome of the Uda River was binned using three different tools (MaxBin 2.0 Wu et al., 2015, MyCC Lin and Liao, 2016, and Busy Bee Web Laczny et al., 2017) prior to dereplication and refinement with the DAS Tool (Sieber et al., 2018). The DAS Tool performs a consensus binning and produces the final bin set. Completeness and contamination rates were assessed using CheckM v. 1.0.12 (Parks et al., 2015) with the “lineage wf” command and default settings. Annotation of the UR-1 genome was carried out using the NCBI Prokaryotic Genome Annotation Pipeline (Tatusova et al., 2016). This genome project has been deposited in the DDBJ/ENA/GenBank under the accession number RXIU00000000.

### Phylogenetic Analysis and Genome Index Calculation

Phylogenomic analyses of *Magnetococcales* genomes were conducted with GTDB-Tk v.0.1.3 using the *de novo* workflow with a set of 120 single-copy marker proteins and the genome taxonomy database (GTDB) (Parks et al., 2018). Concatenated alignments were used to construct a maximum likelihood tree inferred in IQ-Tree using the LG+F+I+G4 model recommended by ModelFinder (Wong et al., 2017), and branch supports were estimated using UFBoot2 (Hoang et al., 2017). The tree was rooted using two *Magnetospirillum* species, Zetaproteobacteria bacterium PCbin4 and Lambdaproteobacteria bacterium PCRbin3 as outgroups.

The MamA, -B, -E, -H, -I, -K, L, -M, -O, -P, -S, and -T amino-acid sequences were independently aligned using MAFFT (Katoh and Standley, 2013), cleaned with Gblocks v. 0.91b (Castresana, 2000) with an option to allow gap positions in the final blocks, and concatenated. These alignments were used to construct a maximum likelihood tree inferred in IQ-Tree using the LG+F+I+G4 model.

The average nucleotide identity (ANI) and average amino acid identity (AAI) were calculated using the ANI/AAI-Matrix online service (Rodriguez-R and Konstantinidis, 2016). Digital DNA-DNA hybridization (dDDH) values were determined using the Genome-to-Genome Distance Calculator (GGDC) 2.1 online software (Meier-Kolthoff et al., 2013). The pairwise percentage of conserved proteins (POCP) was calculated using the script runPOCP.sh (Pantiukh and Grouzdev, 2017; Grouzdev et al., 2018), based on the previously described approach (Qin et al., 2014).

Genes transferred horizontally were identified using the recentHGT program (Li et al., 2018). This program allows the finding of recently occurred events of horizontal gene transfer between closely related species.

### Analyses of Magnetosome Genes and Reconstruction of Metabolic Pathways of Magnetococcales Bacterium UR-1

Computational prediction of CDS and other genomic features, together with functional annotation, was performed using the NCBI automated prokaryotic genome annotation pipeline (https://www.ncbi.nlm.nih.gov/genome/annotation_prok Tatusova et al., 2016). Homologous sequences of magnetosome proteins were identified within the protein database using BLAST searches. The conserved domain structure of putative magnetosome proteins was analyzed with the Batch Web CD-Search Tool with default parameters (Marchler-Bauer et al., 2017). A manual curation of the predicted gene annotations was performed for the metabolic features selected for the current analysis. This was supported by functional analysis with InterProScan and gapped BLAST (Altschul et al., 1997; Quevillon et al., 2005). The metabolic reconstruction was aided by the KEGG Mapper, which included the pathway, BRITE, and MODULE reconstruction tools (Kanehisa et al., 2019).

## Results

### Enrichment of MTB Cells and Diversity Analysis by 16S rRNA Gene

According to the ecological monitoring data, the waters of the Uda River near the sampling site had a pH of 8.04 and a temperature of 11°C. The water body contained low concentrations of phosphorus, as well as ammonium, nitrite, and nitrate ions. The data on physicochemical composition of water samples are summarized in Supplementary Table S1.

**Figure 1:**
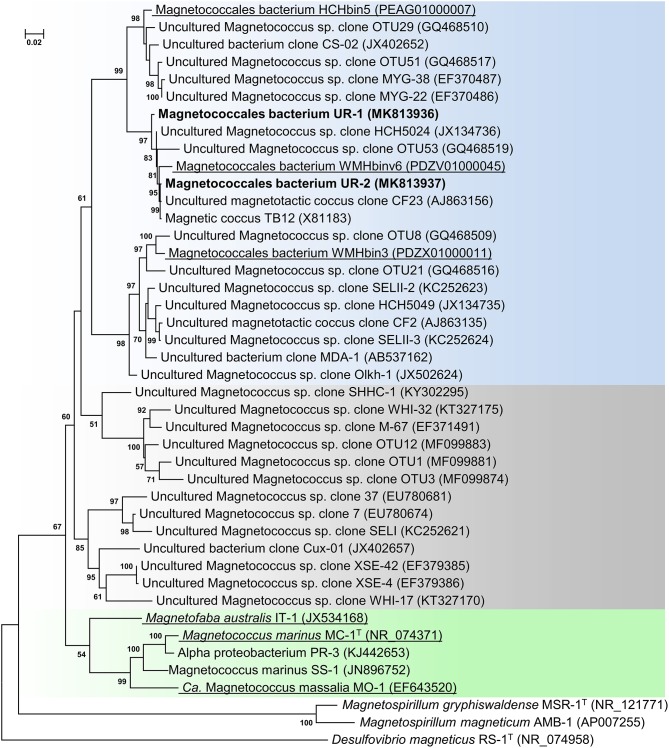
Maximum-likelihood phylogenetic tree based on 16S rRNA gene sequences (1,277 nucleotide sites) reconstructed with evolutionary model GTR+F+I+G4 showing the position of OTUs UR-1 and UR-2 in relation to members of the order *Magnetococcales*. Bootstrap values (>50%) are listed as percentages at the branching points. The scale bar represents nucleotide substitutions per site. The tree was rooted using *Desulfovibrio magneticus* RS-1^T^ as outgroup. Bacteria with genomic sequences are underlined.

Following the magnetic separation, a visible pellet of magnetotactic bacteria had accumulated. Light microscopy examination revealed only motile cocci present in the cell fraction. Microscopy studies using TEM showed that the magnetically concentrated cells contained the dominant group of magnetotactic cocci with characteristically clustered magnetosomes. The clonal library of the Uda microcosm consisted of 71 16S rRNA gene sequences. On the phylogenetic tree, all the obtained sequences formed two OTUs within the order *Magnetococcales*. One OTU, designated as Magnetococcales bacterium UR-1 (hereinafter “UR-1”), was dominant (87% of the library) (Figure 1). The level of similarity was 89.6% for the closest validly described organism *Mc. marinus* MC-1^T^ of the *Magnetococcaceae* family, order *Magnetococcales*. This result suggested that the UR-1 might belong to a new family in the order *Magnetococcales* (Chun et al., 2018).

The phylogenetic tree was constructed using the available 16S rRNA sequences from the MAGs of the freshwater cocci HCHbin5, WMHbinv6, and WMHbin3 (Lin et al., 2018). The level of similarity of UR-1 with WMHbinv6, HCHbin5, and WMHbin3 was 98.2, 96.2, and 93.1%, respectively. The proposed standards for the description of uncultured bacteria are that a level of 16S rRNA sequence similarity of 95–98.6% suggests that analyzed strains belong to the same genus, while 92–95% suggests they belong to the same family (Konstantinidis et al., 2017). Thus, UR-1, HCHbin5, WMHbinv6, and WMHbin3 potentially belong to the same family, within which UR-1, WMHbinv6, and HCHbin5 formed the same genus and WMHbin3 putatively represented another one.

On the phylogenetic tree, the sequence of OTU UR-1 formed a cluster with the sequences HCH5024, CF23, and TB12, which were previously obtained from environmental samples from Germany and China (Spring et al., 1995; Flies et al., 2005b; Wang et al., 2013). The similarity between them was 99%, which indicated their possible affiliation with different strains of the same species or with different very closely related species. The 16S rRNA obtained from the rivers and lakes of Germany, China, and Russia also clustered with the sequences of HCHbin5 and WMHbin3, indicating that the representatives of the putative family, which included OTU UR-1, are widely distributed in freshwater habitats.

### Morphology of UR-1 Cells and Magnetosomes

**Figure 2:**
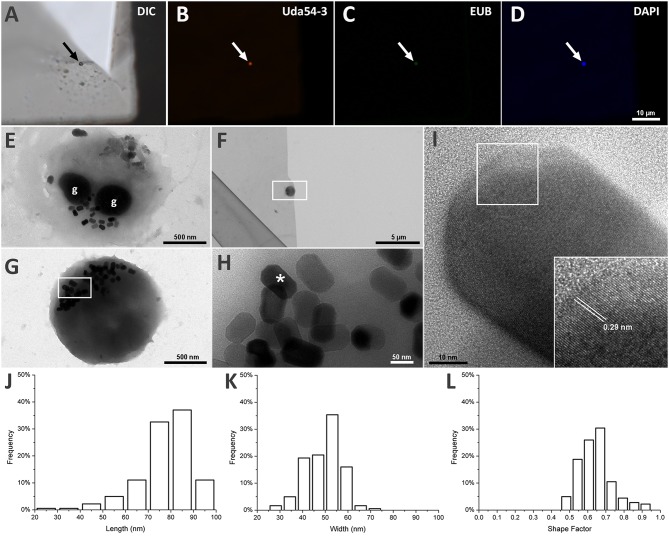
Morphological analysis of strain UR-1 cells and it magnetosomes. **(A)** DIC microscopy of magnetic enriched MTB sample on top of a TEM grid presenting a round cell on the center (arrow). **(B)** Fluorescence microscopy image of the same field capture on A showing the round cell hybridize with probe UR54-3 (arrow). **(C)** Fluorescence microscopy image showing the same cell recognized by probe UR54-3 hybridized with EUB probes (arrow). **(D)** Fluorescence microscopy of the same field showing the round cell stained by DAPI (arrow). **(E)** TEM image of UR-1 cell presenting a coccoid morphology and two electron dense inclusions (g) resembling P-rich granules. **(F)** TEM image of the same coccus present on imaged A by DIC. **(G)** Higher magnification of the area mark by the rectangle on figure **(F)**. The round cell presented magnetosomes not organized in chains and clustered in one side of the cell. **(H)** Higher magnification of the area mark by the rectangle on figure **(G)**. Magnetosomes inside the round cell have an elongated shape, with flat corners. **(I)** HRTEM image of the magnetosome mark with an asterisk on image **(H)**. The insertion shows an interplanar distance of 0.29 nm between the atom columns present on the magnetosome crystal surface, indicating that the crystal is made of magnetite. **(J)** Length frequency distribution of magnetosomes found on UR-1 cells. **(K)** Width frequency distribution of magnetosomes found on UR-1 cells. **(L)** Shape factor frequency distribution of magnetosomes found on UR-1 strain.

Using FISH, we identified a coccoid cell as the individual associated with the 16S rRNA sequence MK813936 retrieved from strain UR-1 sequencing (Figure 2). The specific probe for the strain UR-1 (Uda54-3) hybridized with a round cell (Figures 2A,B), which was also recognized by EUB probes (Figure 2C) and stained by DAPI (Figure 2D). Probe Uda54-3 did not hybridize with other cell types in the sample (Supplementary Figure S1).

The TEM images of the same cell recognized by probe Uda54-3 on Figure 2B are presented in Figures 2F,G and reveal the presence of elongated magnetosomes (Figure 2H); these are not organized in chains but are clustered on **one** side of the cells (Figures 2E,G). The HRTEM of the magnetosomes showed an interplanar distance between the atom columns of 0.29 nm (Figure 2I), a value associated with the oxide of iron magnetite (Martínez-Mera et al., 2007; Zhuang et al., 2015). The magnetosomes of strain UR-1 presented a mean length of 77.4 nm (SD = 11.8 nm, *n* = 181), with more than 90% of the magnetosome length between 65 and 95 nm (Figure 2J), a mean width of 46.2 nm (SD = 7.9 nm), with more than 90% of magnetosomes width between 40.6 and 59.3 nm (Figure 2K), and a mean shape factor of 0.64 (SD = 0.09) with more than 90% of the shape factor value between 0.48 and 0.73 (Figure 2L). Electron-dense inclusions were consistently observed in the UR-1 cell cytoplasm (Figure 2E). These structures have been observed in most magnetotactic cocci characterized so far (Dziuba et al., 2013; Abreu et al., 2014) and are correlated with polyphosphate (poly-P) inclusions (Lins and Farina, 1999; Keim et al., 2005).

### MAG Statistics

The assembly and binning of data resulted in the MAG of magnetotactic coccus UR-1. It consisted of 546 contigs, with a total length of about 4143644 bp. The GC composition was 52.2 mol%. In accordance with the standards imposed on metagenomic assemblies, the genome of UR-1 had a high quality (completeness > 90%, contamination < 5%) (Supplementary Table S2). In the assembled genome, the 16S rRNA sequence was identified in the contig with a length of 25,034 bp and was identical to the sequence of the dominant OTU UR-1. The specific probe Uda54-3 used for FISH-TEM morphology identification of OTU UR-1 also matched the 16S rRNA of the genome, thereby confirming the link between the genome of the coccus UR-1 and the identified cell phenotype.

### Delineation of the “*Ca*. Magnetaquicoccaceae” Family Within the Class *Magnetococcales*

**Figure 3:**
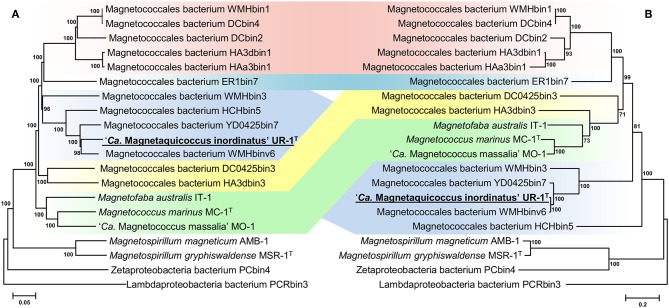
Maximum-likelihood phylogenetic trees inferred from a comparison of **(A)** concatenated *Magnetococcales* 120 single copy marker proteins showing the position of the UR-1 and **(B)** concatenated magnetosome associated protein (MamABEHIKLMOPST) sequences. Both trees reconstructed with evolutionary model LG+F+I+G4. The scale bar represents amino acid substitutions per site.

**Figure 4:**
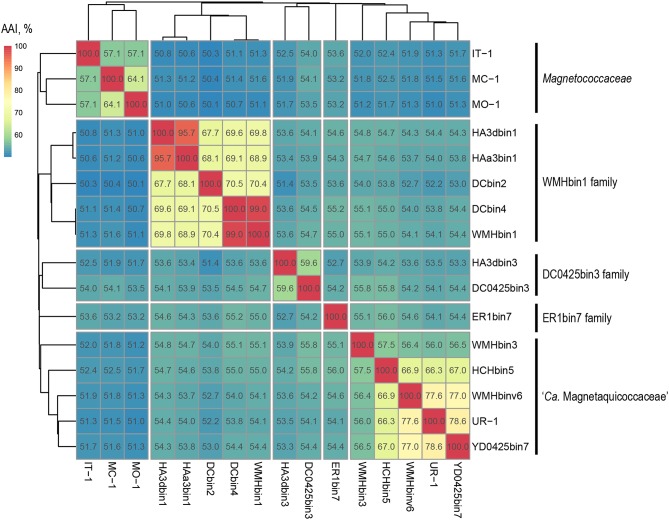
Whole-genome relationships within the order *Magnetococcales*. The heat map shows pairwise comparisons of AAI between genomes of the *Magnetococcales* strains. Thermal color scale located at the upper left corner. Boxed regions indicate inferred family clusters based on AAI comparisons. Black lines located at the right indicate a proposed family name.

Separation of taxa requires the establishment of permissible criteria. Phylogeny based on the genomic sequence is considered to be the main tool (Lang et al., 2013; Chun et al., 2018). The current requirement is that branches must (i) be monophyletic (Rossello-Mora and Amann, 2001) and (ii) result in minimum changes in the current taxonomy (Orata et al., 2018). On the phylogenomic tree, the *Magnetococcales* genomes formed five clades that were supported by high bootstrap values (100%) (Figure 3A). UR-1 formed a clade together with WMHbinv6, YD0425bin7, HCHbin5, and WMHbin3, which correlated with the 16S rRNA phylogenetic tree. Cultured marine strains MO-1, IT-1, and MC-1^T^ of the *Magnetococcaceae* family formed a separate branch, consistent with the topology on the 16S rRNA tree. The third group consisted of five genomes: DC0425bin3, WMHbin1, DCbin4, HAa3bin1, HA3dbin1, and DCbin2. The fourth branch was formed solely by ER1bin7, and the fifth group included the genomes DC0425bin3 and HA3dbin3. The five identified clades may represent five families within the order *Magnetococcales*. These groups were preliminarily designated as “*Magnetococcaceae,”* “UR-1,” “WMHbin1,” “ER1bin7,” and “DC0425bin3.” These lineages were also confirmed by the analysis available in Genome Taxonomy Database (GTDB), except for “ER1bin7” (Parks et al., 2018). We checked whether the identified clades could represent candidate families by conducting additional studies on the available *Magnetococcales* genomes. The taxonomic ranks at the family and genus levels were separated using numerical indices based on the amino acid sequences of the genome, AAI and POCP. The AAI analysis showed identity values ranging from 50.1 to 99.0% between all analyzed genomes (Figure 4). The AAI value between the representatives of the identified phylogenetic branches was 50.1 to 55.8%. As the previous studies have shown, AAI has no clear boundaries for taxon separation (Konstantinidis and Tiedje, 2005; Luo et al., 2014). According to Konstantinidis et al. (2017), representatives of the same family may have AAI values of 45–65%, and representatives of the same genus may have values of 65–95%. The use of the lower boundary of 45 and 50% would lead to the unification of all *Magnetococcales* genomes into one family, but the use of values of 55–56% for the separation of families perfectly correlated with the branching of the phylogenomic tree and confirmed the possibility of separating five families within the order *Magnetococcales*. If the lower value of 65% is applied for the separation of genera, the cultivated representatives of the genus *Magnetococcus*, MC-1^T^ and MO-1, would belong to different genera. However, this contradicts the current taxonomy. Thus, setting the limit of differentiation of close genera at 64–65% confirmed the established separation of the genera *Magnetofaba* and *Magnetococcus*. When we applied the same criterion to the “UR-1” group, two genera could be identified: the first included UR-1, YD0425bin7, WMHbinv6, and HCHbin5, and the second genus was formed by strain WMHbin3. Similarly, within the clade “WMHbin1,” all representatives formed a single genus, and in the clade DC0425bin3, two genera were distinguished. The proposed AAI ranges can be applied to the constantly increasing number of available genomes of the class “*Ca*. Etaproteobacteria,” which would enable consistent and reliable classification at the genus and family levels of the sequences derived from axenic cultures or metagenome analyses in the future.

POCP is another tool for differentiating between genera, and a fixed genus boundary of 50% of conserved proteins has been proposed (Qin et al., 2014). The POCP analysis showed that the values between all cocci ranged from 32.9 to 90.3% (Supplementary Table S3). In general, the POCP results supported the AAI results; however, several cocci had low values of genome completeness, which strongly influences the final result of the POCP calculation and can thus lead to unreliable results. Interestingly, the POCP values between representatives of the two genera *Magnetofaba* and *Magnetococcus* were 53–57%, which is higher than the proposed genera separation threshold at 50% (Qin et al., 2014). Thus, a 50% POCP threshold is not applicable for separating genera within the order *Magnetococcales*. A similar situation was observed for the differentiation within other taxonomic groups, such as the families *Methylococcaceae* (Orata et al., 2018) and *Neisseriaceae* (Li Y. et al., 2017).

The strains were distinguished at the species level using the nucleotide sequence-based indexes ANI and dDDH. The ANI analysis showed identity values ranging from 70 to 99.4% between all *Magnetococcales* genomes (Supplementary Table S4). The ANI values for strain UR-1 were below the standard species separation threshold (<95–96%) with all analyzed sequences, indicating that it belongs to a novel species (Goris et al., 2007). The dDDH analysis showed values ranging from 15.1 to 93.3% (Supplementary Table S5). The dDDH values for strain UR-1 were also below the standard species separation threshold (<70%) with all analyzed sequences, thereby confirming the results of the ANI calculation (Auch et al., 2010). According to the results of ANI and dDDH, within the clade “WMHbin1,” the strains DCbin4 and WMHbin1 belonged to the same species, whereas the values between HA3bin1 and HAa3bin1 (95.3%) are at the boundary for delineation of two species (95–96%); hence, they could not be separated with confidence.

Based on the results obtained by analyzing genome sequences, we propose a delineation of five candidate families within the order *Magnetococcales* and differentiate strain UR-1 as a novel candidate species within one of these families, for which we propose the name “*Ca*. Magnetaquicoccaceae.” For strain UR-1, we propose the name “*Ca*. Magnetaquicoccus inordinatus.”

### Genome Analysis of the “*Ca*. Magnetaquicoccus inordinatus” UR-1

#### Magnetosome Genes

**Figure 5:**
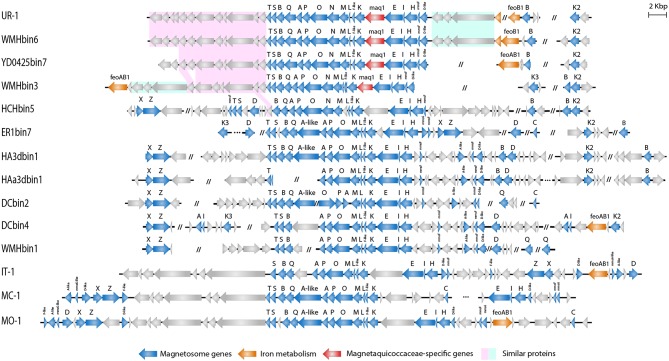
Organization of MAI region in genomes of *Magnetococcales* strains.

The assembly of nearly the complete genome of UR-1 allowed identification of parts of the magnetosome genomic island (MAI). Most of the magnetosome synthesis genes were found in the contig RXIU01000008 (44,671 bp). This contig contained 42 genes, 17 of which had a high level of similarity with the *mam* and *mms* genes from the representatives of *Magnetococcaceae* and magnetotactic *Alphaproteobacteria* (Supplementary Table S6). In addition, the second *mamK* gene was found in the contig RXIU01000185 (7,100 bp). The *mamAB* cluster contained the *mamK, mamF-*like*, mamL, mamM, mamN, mamO, mamP, mamA, mamQ, mamB, mamS*, and *mamT* genes (Figure 5). A separate *mamHIE* cluster was also found, and the *mmsF*-like and *mamD*-like genes were located upstream of that. Interestingly, MmsF-like and MamD-like had a high level of similarity with the homologous proteins found in “*Ca*. Terasakiella magnetica” PR-1 (Monteil et al., 2018) and *Magnetospirillum caucaseum* SO-1^T^ (Dziuba et al., 2016) (55 and 50%, respectively). Between the *mamAB* and *mamHIE* clusters, we identified an ORF, the putative product of which has no predicted function and homology with any of the proteins in the GenBank database. At the end of the contig in which the MAI was located, an incomplete gene was found that has a high similarity to *feoA1*. The location of this part of the gene was similar to that of the freshwater coccus WMHbinv6, which has *feoAB1* genes 7.5 kb upstream of MAI. An incomplete *feoB1* gene was also found in UR-1 (contig RXIU01000148), next to which there was a gene with a high similarity to *mamB*.

A subsequent phylogenetic analysis showed that the tree based on the concatenated amino acid sequences of the Mam protein was non-collinear with the tree developed on the basis of core genome proteins. On the phylogenetic tree of the magnetosome proteins, UR-1 clustered together with WMHbinv6, UD0425bin7, and WMHbin3 (Figure 3B); however, the clade was external to the branches formed by other strains. In addition, the concatenate of HCHbin5 formed a separate branch and did not cluster with representatives of the same genera. The inconsistency between positions of branches on the obtained trees suggested the horizontal transfer (HGT) of magnetosome genes in “*Ca*. Magnetaquicoccaceae” and within the family, in HCHbin5. We also provided a comparison of phylogenetic trees based on concatenated protein sequences from *mamHIE* and *mamAB* clusters (Supplementary Figure S2). Both trees were mostly congruent, with the exception of HCHbin5 and DC0425bin3 positions. However, the tree of MamHIE proteins had low branch support; therefore, solving the question of how magnetosome clusters evolved in these two strains will require further genome sequences of “*Ca*. Etaproteobacteria” are needed. In spite of this, the result of the tree topology comparisons suggested that the evolutionary history of both clusters was largely the same in the “*Ca*. Etaproteobacteria” strains.

**Figure 6:**
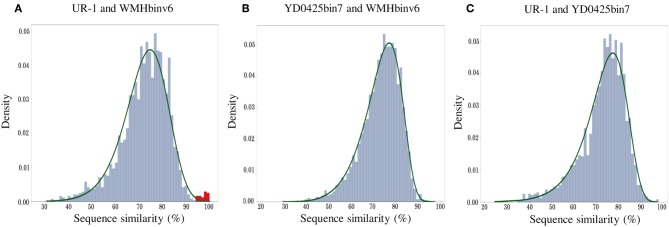
Histograms of sequence similarity values between **(A)** UR-1 and WMHbinv6, **(B)** YD0425bin7 and WMHbinv6, **(C)** UR-1 and YD0425bin7. The x-axis and y-axis represent the values of sequence similarity and density, respectively. The green line shows the boundaries of the Weibull distribution. The distribution of sequence similarity of horizontally transferred genes is highlighted in red.

In addition to the general non-collinearity of the trees, an interesting result was observed. The phylogenomic tree shows (Figure 3A) that the amino acid substitution rate between strains UR-1 and WMHbinv6 is much higher than that on the phylogenetic tree of the magnetosome proteins (Figure 3B). This discrepancy in branch lengths may be due to recent horizontal gene transfer (Syvanen, 1994; Koonin et al., 2001). The putative horizontal gene transfer was explored using the recentHGT tool, which detects HGT events at the genome level between closely related species (Li et al., 2018). The strain YD0425bin7, closely related to UR-1 and WMHbinv6, was also included in the analysis. The recentHGT strategy allowed calculation of WMHbinv6 and YD0425bin7 sequence similarity values for all homologous genes from strains UR-1. The analysis of the obtained data revealed that all values between strains YD0425bin7 and WMHbinv6, as well as strains UR-1 and YD0425bin7, complied with the Weibull distribution (Figures 6B,C, respectively). Thus, no signs of recent HGT events were detected between these two pairs of strains. However, in case of UR-1 and WMHbinv6, the similarity values of the magnetosome-associated genes did not fit the Weibull distribution (Figure 6A, Supplementary Table S7). The similarity values of the detected genes were 95–100%, which is much higher than the similarity between the housekeeping genes. Thus, the obtained results indicated that MAI genes in UR-1 and WMHbinv6 had undergone a recent HGT event.

Comparison of the magnetosome island organization of UR-1 with the other strains demonstrated that representatives of the proposed family “*Ca*. Magnetaquicoccaceae,” namely UR-1, WMHbinv6, YD0425bin7, WMHbin3, and HCHbin5, had a similar set of magnetosome biomineralization genes. Thus, the *mamN* gene was included in the *mamAB* cluster in all of them, unlike in the other *Magnetococcales* strains. When searching for the closest homologs for the MamN protein, we identified a high level of similarity with *Magnetovibrio blakemorei* (43.89%) (Trubitsyn et al., 2016) (Supplementary Table S6). The *mamCXZ* gene cluster, which is present in the genomes of the *Magnetococcaceae* strains and the other three proposed families, was not found in the UR-1 genome or in the closest related strains WMHbinv6, YD0425bin7, and WMHbin3. The *mamA*-like gene, which is located inside the *mamAB* cluster, and the *mms6* genes were also absent in genomes of UR-1 and related strains. An additional difference was the presence of a gene located between *mamK* and *mamE*. The hypothetical protein encoded by that gene had no homology with any of the proteins in the GenBank database and is probably specific to members of the family “*Ca*. Magnetaquicoccaceae.” That gene was designated *maq1* (“Magnetaquicoccaceae” specific).

Three other genes encoding proteins with unknown functions were found between the *mamD*-like and *feoA1* genes in the UR-1 genome. As predicted by COG and pfam, protein WP_130470120 has an EH signature domain, WP_130470121 was classified as flagellar motor protein MotB, and WP_130470122 contains apolipoprotein A1/A4/E domains. These proteins did not have homology with any of the known proteins of MTB but were also present in WMHbinv6 and WMHbin3. The closest homologs were identified in non-magnetotactic *Gamma-* and *Deltaproteobacteria*. The organization and arrangement of these three genes was identical in UR-1 and WMHbinv6; this cluster was 9 kb downstream of the *mamT* gene in WMHbin3, and the *feoAB1* genes were located next to them. Another similarity in MAI organization between UR-1 and WMHbinv6 was observed for 11 genes located downstream of *mamT*. Eight and seven of them, in the same order, were also found in the strains closest to the UR-1, namely YD0425bin7 and WMHbin3, respectively. Their products are associated with hypothetical and chemotaxis proteins. At the same time, HCHbin5, which belongs to the same candidate genus as UR-1, differed significantly in the MAI structure from the other members of the same group. Another set of the genes flanked the main cluster of MAI, and additional hypothetical proteins were present in the HCHbin5 *mamAB* cluster. Furthermore, in contrast to the other strains from the proposed family “*Ca*. Magnetaquicoccaceae,” the *mamXZ* genes were found in the HCHbin5 genome. In addition, the gene between *mamK* and *mamE* in the MAI of HCHbin5 had a low similarity (5% coverage) with the *maq1* gene of “*Ca*. Magnetaquicoccaceae.”

In conclusion, a similar set and organization of magnetosome genes in strains UR-1, WMHbinv6, YD0425bin7, and WMHbin3 may indicate that the strains synthesize magnetosomes with a similar arrangement, whereas the organization of magnetosome genes in HCHbin5 significantly differed from those of the other members of the family “*Ca*. Magnetaquicoccaceae.” This suggests that magnetosomes might be arranged differently in this strain.

### Metabolic Reconstruction

**Figure 7:**
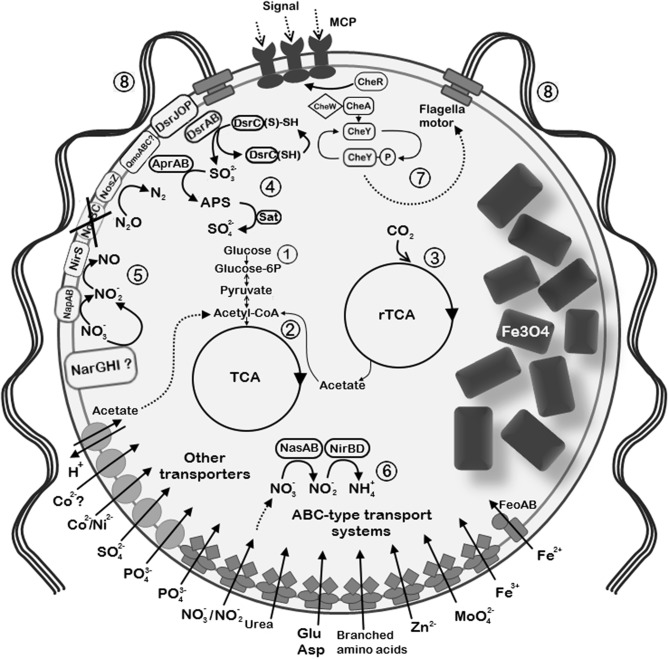
Reconstruction of the selected metabolic and structural features of “*Ca*. Magnetaquicoccus inordinatus” based on genome analysis and TEM micrographs. The following metabolic pathways are emphasized: (1) glycolysis and glyconeogenesis; (2) tricarboxylic acid cycle (TCA); (3) autotrophic CO_2_ fixation through reverse TCA (rTCA); (4) sulfide oxidation trough reverse Dsr (dissimilatory sulfite reductase) pathway. Sulfite which is formed via Dsr operation is further oxidized to sulfate through the intermediate 5'-adenylyl sulfate (APS) by APS reductase (AprAB) and sulfate adenylyltransferase (Sat). (5) Dissimilatory nitrate reduction is possible through the periplasmic nitrate reductase (NapAB) and putative membrane-bound nitrate reductase complex (NarGHI). Nitrite reduction is further possible by nitrite reductase NirS. Since *norBC* genes were not found (crossed out), might be not capable of nitric oxide reduction, whereas nitrous oxide can be reduced to nitrogen by nitrous oxide reductase (NosZ). (6) Assimilatory nitrate and nitrite reduction in the cytoplasm through nitrate reductase (NasA) and nitrite reductase NirBD, respectively. (7) Chemotaxis complex is represented by methyl-accepting chemotaxis proteins (MCP) and Che proteins. (8) The species presumably synthesizes multiple flagella due to the presence of high number of flagellin genes. Since two bunches of flagella are common among magnetotactic cocci, similar organization is assumed in “*Ca*. Magnetaquicoccus inordinatus”.

Recovering of the nearly complete genome of UR-1 allowed prediction of the metabolic traits of this novel magnetotactic strain. Comparison of the UR-1genome with the MAGs of other freshwater *Magnetococcales* allowed the determination of group-specific metabolic features that support the proposed division of the studied strains into distinct phylogenetic groups. Here, we focus on selected metabolic pathways, including carbon fixation, and nitrogen, sulfur, and phosphorus metabolism. The description of the cellular transport, oxidative stress defense strategies, chemotaxis, and motility of UR-1 is provided in Supplementary File 1. The metabolic features of the novel strain are summarized in Figure 7.

### Carbon Fixation

Strong experimental evidence supports the utilization of a reverse tricarboxylic acid cycle (rTCA) for CO_2_ fixation by MC-1^T^ and IT-1 (Williams et al., 2006; Araujo et al., 2016). The genome of MC-1^T^ contains at least three enzymes key to the rTCA: 2-oxoglutarate:acceptor oxidoreductase, pyruvate:acceptor oxidoreductase, and fumarate reductase. However, although citrate cleavage to acetyl-CoA and oxaloacetate is detected in this organism, none of the genes for citryl-CoA synthetase, citryl-CoA lyase, or the *bona fide* ATP:citrate lyase (ACL) were found in its genome. Nevertheless, two ORFs, which encode subunits of a postulated type II ACL (Mmc1_3638, Mmc1_3639), are present in MC-1^T^ (Schübbe et al., 2009; Hügler and Sievert, 2011). The same is true for the genomes of other cultivated marine magnetotactic cocci “*Ca*. Mc. massalia” MO-1 and *Mf. australis* IT-1, suggesting that this group of bacteria employs another type of enzymes for catalyzing citrate cleavage. Our analysis showed that the presence of the genes for the putative type II ACL and the other key enzymes appears to be shared by UR-1 and many other freshwater magnetotactic strains (Supplementary Table S8), implying that rTCA may be a common trait for this group. However, in five MAGs (WMHbin1, DCbin2, DCbin4, HA3dbin1, and HAa3bin1), none of the subunits of the putative ACL were found, while the other key proteins of the pathway are present. This might reflect an incomplete state of the genomes. Interestingly, these strains form a separate group at the family level (Figure 3), which may hint that some other mechanism of carbon fixation, if any occurs, might be intrinsic to this group.

### Nitrogen Metabolism

The genome of UR-1 contains the entire set of *nif* genes for di-nitrogen fixation, a feature that is frequently encountered among MTB, with only a few known exceptions, i.e., *Magnetospira sp*. QH-2 and Gammaproteobacterial magnetotactic strain SS-5 (Lefèvre et al., 2012b; Ji et al., 2014). The *nif* cluster in UR-1 comprises 18 genes, including *nifVXNEBQHDKTY* with the nitrogen fixation transcriptional regulator *nifA* localized separately, approximately 7 kb downstream of *nifY*. The *nifU* gene, which is proposed to have a redox function for assembly of the Fe-S cluster in nitrogenase complex, was also found and is located at a different chromosomal locus (Agar et al., 2000). Unlike the case for MC-1^T^ and MO-1, the *nif* operon in UR-1 is lacking the gene *nifZ*, and no homologs could be found outside the *nif* gene cluster. NifZ is known to play an accessory, but not essential, role in the maturation of MoFe nitrogenase P-clusters (Jimenez-Vicente et al., 2019). Interestingly, *nifZ* could not be found in the *nif* operons of several other uncultivated freshwater *Magnetococcales* strains, which otherwise have essential *nif* genes: YD0425bin7, WMHbin3, WMHbin1, DC0425bin3, DCbin4, and HA3dbin1. We cannot rule out that *nifZ* does not constitute a part of the *nif* operon in these strains; it could be missing due to sequence incompleteness. Alternatively, other mechanism of maturation of the nitrogenase complex could have evolved in these groups of bacteria.

UR-1 is potentially capable of assimilatory and dissimilatory nitrate reduction. The *nap* operon, which encodes periplasmic nitrate reductase (NAP) together with the genes necessary for its maturation, comprises 6 genes, i.e., *napDAGHBC*. Interestingly, the *nap* operon in UR-1 is lacking the gene for the non-heme iron-sulfur protein NapF, which is the first gene in this operon in the majority of denitrifying bacteria, including MC-1^T^ and MO-1. However, in *E. coli*, NapF is not essential for either catalytic activity or for maturation of the NAP complex, implying that UR-1 can still possess a fully active NAP (Potter and Cole, 1999). In addition to NAP, an operon encoding alpha chain (molybdopterin-containing catalytic subunit), beta chain (iron-sulfur center-containing electron transfer unit) and gamma chain (heme b) of a complex protein belonging to membrane-bound nitrate reductase NarGHI-like anaerobic respiratory enzymes is present in the genome of UR-1 and the closely related freshwater strains WMHbinv6 and YD0425bin7. The gene for a protein with high similarity to TorD-like chaperones, which mediate maturation of the complexes of the molybdopterin respiratory enzymes, was also determined in UR-1 in close proximity to the putative *nar* operon, suggesting a probably complete assembly and hence functionality of the NarGHI-like enzyme. However, the relatively low similarity of these proteins from UR-1 to the well-studied NAR from other bacteria (e.g., 26.1% for catalytic subunit NarG compared to that of *E. coli*) does not allow us to predict the type of reduced substrate with confidence, as several other anaerobic respiration oxido-reductases have very similar sequences (e.g., dimethyl sulfoxide, selenate, and chlorate reductase) (Leimkühler and Iobbi-Nivol, 2015). This may suggest an extended ability of UR-1 and related strains to utilize alternative terminal electron acceptors.

The presence of genes for NirBD and NirS in the genome of UR-1 suggests its ability to further reduce nitrite produced by nitrate reductases to ammonia and nitric oxide, respectively. Interestingly, two genes for *cd*_1_ cytochrome NO-forming nitrite reductase, NirS, were found in the genome of UR-1. One of the *nirS* genes (contig RXIU01000046) is followed by *nirCFGLHJ*, a set of genes encoding the important factors for the biogenesis of the *d*_1_ cofactor of NirS. As the gene cluster is located at the end of the contig having *nirJ* lopped approximately in the middle, determining a precise organization of the *nir* operon is impossible in UR-1. The gene order in the *nir* operon may vary among the denitrifying bacteria, with the usually conserved *nirFDLGH* set and *nirN* localized as the last genes in the operon (Zajicek et al., 2009). In UR-1, a gene fragment belonging to the putative *nirN* is localized at the edge of contig RXIU01000360, suggesting that contigs RXIU01000046 and RXIU01000360 may be linked together. Thus, UR-1 possesses almost the entire set of genes needed for the synthesis and functional assembly of nitrite reductase NirS, lacking only the *nirE* gene. The *nirE* gene is almost always found adjacent to *nirS*, and only a few exceptions are known (e.g., *Aromatoleum aromaticum*) where it is located in a separate operon (Zajicek et al., 2009). Hence, if *nirE* is localized outside the *nir* operon in UR-1, it quite possibly was not covered by sequencing.

The second *nirS* gene is located in contig RXIU01000354, adjacent to a gene for a NirC-like cytochrome. Due to the incomplete state of the genome, we cannot rule out that UR-1 has a second set of the genes for NirS biosynthesis as well that could not be identified in the genome at present. Redundancy in *nirS* genes may indicate the importance of nitrite respiration in the energy conservation of UR-1.

Curiously, the structural genes for nitric oxide reductase, *norBC*, were not found in the genome of UR-1 and the related strains WMHbinv6 and HCHbin5 (YD0425bin7 differed, as it had the full set of *nor* genes, Supplementary Table S8). At the same time, *norQ* and *norD*, which are usually parts of the *norBCQD* operon, can be still found in the genome of UR-1, closely adjacent one to another. The absence of *norBC* might be explained by the incomplete state of the UR-1, WMHbinv6, and HCHbin5 genomes. Although truncated versions of denitrification are frequent in bacteria from various environments, the lack of nitric oxide reduction is relatively rare due to the danger of accumulation of highly toxic NO (Lycus et al., 2017). Finally, UR-1 appears to be capable of converting N_2_O to N_2_ in the last step of denitrification, owing to the presence of the *nosZDFYL* operon.

In contrast to the marine cocci (e.g., MC-1^T^, MO-1, and IT-1), UR-1 has the potential ability for assimilatory nitrate reduction as it has the gene set for NADH-dependent assimilatory nitrate and nitrite reductases in its genome. The structural gene for assimilatory nitrate reductase, NasA, was found adjacent to the genes for nitrite reductases *nirD* and *nirB* in an operon-like structure, and was followed by genes for the ABC nitrate transporter *nrtABC* (see section “Transport” in Supplementary File 1), two-component transcriptional regulator *nasTS*, an HPP-family protein of unknown function, and a fragment of the MFS transporter gene. Interestingly, the presence of nitrate assimilation genes is not common among the available genomes of uncultivated freshwater *Magnetococcales* strains, being restricted to UR-1 and the closely related WMHbinv6.

### Sulfur Metabolism

The genes for the key enzyme of sulfate assimilation, sulfate adenylyltransferase *cycN*, and *cycD*, as well as 3'-phosphoadenosine 5'-phosphosulfate synthase PAPSS, were not found in the genome of UR-1 and the other freshwater *Magnetococcales* strains. This consistence suggests that these genes are more likely to be missing from the analyzed genomes, rather than not being covered by sequencing. The predicted inability to assimilate sulfate by these organisms is consistent with the absence of specialized transport systems for sulfate, which usually occur in bacteria capable of sulfate assimilation (Hryniewicz and Kredich, 1991). Considering that MTB inhabit anoxic and oxic-anoxic transition zones in the aquatic sediments, which are typically rich in reduced sulfur compounds, these organisms should be able to dispense with assimilatory sulfate reduction.

Our analysis predicts that reduced sulfur plays an important role in the metabolism of UR-1, presumably serving as substrate for dissimilatory sulfur oxidation (and possibly reduction as well). MC-1^T^ is well-established as capable of oxidizing thiosulfate or sulfide when it grows chemolithoautotrophically. MC-1^T^ contains a truncated minimalistic *soxXYZAB* operon and additional putative *sox* genes distributed in the chromosome in three distinct regions (Schübbe et al., 2009; Bazylinski et al., 2013). The lack of the sulfur dehydrogenase genes *soxCD* is consistent with accumulation of sulfur deposits in MC-1^T^ (Dahl, 2015). In contrast to marine magnetic cocci, most of the essential *sox* genes were not found in the genome of UR-1 and other available genomes of “*Ca*. Etaproteobacteria,” with the exception of HA3dbin3. At the same time, genes for the periplasmic protein SoxYZ were found in almost all the genomes. The fact that *soxXYZAB* genes usually constitute a single operon and the consistent absence of all *sox* genes except *soxYZ* from the analyzed genomes suggest that most of the freshwater magnetotactic cocci more likely lack the SOX pathway, rather than indicating that the *sox* genes were not covered by sequencing.

The question arises whether the *soxYZ* genes in these bacteria are non-functional remnants of the complete *sox* operon or whether they play a role in a different process. A recent study by Dahl et al. demonstrated that SoxYZ alone is involved in sulfite oxidation in *Allochromatium vinosum*, while the other Sox proteins did not appear to play any role in this process (Dahl et al., 2013). Therefore, in freshwater *Magnetococcales*, SoxYZ may contribute to the oxidation of sulfite produced by the reverse-acting DsrAB, in parallel with the adenylylsulfate reductase in the 5'-adenylyl sulfate (APS) sulfite oxidation pathway (see below).

UR-1 and the other analyzed strains may be potentially capable of sulfide oxidation through a flavocytochrome *c* sulfide dehydrogenase (FccAB). That protein consists of a large flavoprotein (FccB) and a smaller cytochrome *c* (FccA) subunit, which are related to SoxF and SoxE, respectively (Sander and Dahl, 2009). Sulfide oxidation catalyzed by FccAB is expected to form sulfur globules, but none were observed in the available TEM micrographs of UR-1. Sulfur from deposits can be oxidized to sulfite through a reverse-acting dissimilatory sulfite reductase DsrAB, which has an indispensable role in the oxidation of sulfur globules in *A. vinosum* (Sander and Dahl, 2009). Interestingly, UR-1 contains two copies of the *dsrABL* genes and a single copy of each *dsrC* and *dsrFEHJOP* gene cluster. The presence of *dsrEFH* and *dsrL* indicates the reverse (i.e., sulfur oxidizing) type of Dsr pathway in UR-1 and related bacteria (Ghosh and Dam, 2009). This is also confirmed by the phylogenetic analysis, which showed clustering of the dsrAB genes from UR-1 within the sulfur-oxidizing group (Supplementary Figure S3). They formed a monophyletic branch with sulfur-oxidizing *Chlorobi* and *Mc. Marinus*, which was consistent with a previous study (Müller et al., 2015). The second copy of DsrAB of the *Magnetococcales* members generally belongs in the sulfur-oxidizing group, but it formed a separate monophyletic branch. Due to their distant position and the absence of a cultured strain with a known type of sulfur metabolism, the type of the second DsrAB in *Magnetococcales* requires further confirmation.

In contrast to the marine MTB (e.g., MC-1^T^ and MO-1), UR-1 and other related strains appear to have a potential to further oxidize sulfite produced in dissimilatory sulfur oxidation by indirect AMP-dependent oxidation via the intermediate adenylsulfate (APS). In the reverse direction, this pathway can be also used during dissimilatory sulfate reduction (Matias et al., 2005). Therefore, we cannot rule out that the strains are also capable of respiratory sulfate reduction employing the same enzymes. The APS reductase AprAB catalyzes the formation of APS from sulfite and AMP. In a second step, the AMP moiety is transferred to pyrophosphate by ATP sulfurylase (ATP:sulfate adenylyltransferase) or to phosphate by adenylylsulfate:phosphate adenylyltransferase (Sander and Dahl, 2009). Similar to the genes in some other sulfur-oxidizing bacteria, the genes for ATP sulfurylase (*sat*) and APS reductase (*aprAB*) are encoded in the same operon in UR-1 (RXIU01000214). However, the adenylylsulfate:phosphate adenylyltransferase was not found in the genome of UR-1.

In addition to the genes discussed in this section, other gene clusters potentially having accessory functions in sulfur metabolism were found in UR-1. Their descriptions and speculations as to their potential functions are presented in the Supplementary File 1.

### Phosphorus Metabolism

Phosphate (P_i_) is an essential nutrient that is often scarce in the natural environment. Bacteria take up P_i_ by low-affinity inorganic phosphate transporters (e.g., PitAB in *E. coli*), as well as by the high-affinity ABC-type phosphate-specific transporter Pst. Many bacteria are capable of employing the ABC transport system PhnCDE for phosphonates, the compounds containing carbon-phosphorus (C-P) bonds (Villarreal-Chiu et al., 2012). The genomes of the marine magnetotactic cocci include both low-affinity and high-affinity transporters for P_i_, as well as the high-affinity PhnCDE for phosphonates, which reflects adaptation of these organisms to the phosphorus limitation common in some marine habitats (Tyrrell, 1999; Schübbe et al., 2009). Multiple (up to 5) low-affinity Pit-like phosphate transporters were found in the genome of UR-1 and the closely related genomes WMHbinv6, YD0425bin7, and HCHbin5. Genes for these transporters were also revealed in WMHbin3 and ER1bin7, but not in the other available MAGs (Supplementary Table S8). The genome of UR-1 contains all the essential genes for the Pst P_i_–specific transporter (*pstD, A, B, S*), as well as for the phosphorus uptake specific transcriptional regulator PhoU. Interestingly, the presence of several copies of these gene clusters appears to be common among freshwater *Magnetococcales* strains (Supplementary Table S8). In contrast to marine magnetotactic cocci, no phosphonate uptake systems were found in the analyzed freshwater strains. In UR-1 and several other analyzed genomes, genes similar to those for the putative phosphonate-binding periplasmic protein PhnD were found, but the other components of the transporting system were absent, suggesting that these are non-functional remnants or have a different function.

Accumulation of polyphosphate (poly-P) granules is common among MTB, such as MC-1^T^ and *Magnetospirillum* spp. The TEM micrographs of UR-1 also revealed prominent dark inclusions, reminding poly-P of other bacteria (Figure 2E) (Lins and Farina, 1999; Keim et al., 2005). Poly-P kinase (PPK) is a principle enzyme that catalyzes the transfer of the terminal phosphate of ATP (PPK1) or of GTP (PPK2) to an active site of the protein, the initial step in the progressive synthesis of a long poly-P chain. The reaction is reversible, and in case of PPK2, it is used to synthesize GTP from poly-P (Rao et al., 2009). Many bacteria possess both types of PPK, whereas only one type is found in others. The exclusive presence of genes for PPK2, but not for PPK1, appears to be an intrinsic feature of both marine and freshwater *Magnetococcales* strains. Interestingly, usually several genes for PPK2 were present. In UR-1, two genes for PPK2 (WP_130472334.1 and WP_130472795.1) shared 49.4% identity with each other. The function of the synthesis and utilization of poly-P may be divided between different PPK2 enzymes in UR-1. Bacteria capable of synthesizing poly-P evolved several mechanisms for efficient poly-P utilization. The enzyme catalyzing the incorporation of P_i_ into AMP to restore triphosphate, poly-P:AMP phosphotransferase PAP, appears to be absent from the genomes of most *Magnetococcales*, except for *Mf. australis* IT-1. At the same time, the marine magnetic cocci, as well as many genomes of freshwater *Magnetococcales* strains, possess a gene for exopolyphophatase (PPX), which product hydrolyses and progressively releases the terminal phosphates from linear poly-P. Moreover, a high transcription level of the *ppx* gene was demonstrated in a group of marine magnetotactic cocci that accumulate conspicuous poly-P inclusions and seem to serve as phosphate shuttles within the marine suboxic zone (Schulz-Vogt et al., 2019). Intriguingly, no known genes for PPX were found in UR-1 and the related genomes, WMHbinv6, YD0425bin7, and HCHbin5. Although we cannot rule out that the corresponding genes had not been covered by sequencing, this consistent absence of them in all genomes of the strains closely related to UR-1 suggests that the ability to use poly-P in these organisms might be limited to the predicted GTP synthesis by a PPK2-like enzyme.

## Discussion

This paper is the first to attempt to link the cell structure and magnetosome characteristics of an uncultivated freshwater magnetotactic coccus with a high quality MAG using FISH-TEM.

As demonstrated by TEM analysis, strain UR-1 is remarkable for its synthesis of clustered magnetosomes instead of the ordinary magnetosome chains observed in cultivated MTB and most of the uncultured MTB. The magnetosome formation and arrangement is strictly controlled genetically in the model magnetotactic organisms of the genus *Magnetospirillum*; therefore, we can assume that the peculiar organization of magnetosomes in UR-1 is also determined by the magnetosome gene clusters revealed in this study. The chain formation in *Magnetospirillum* is mediated by the *mamK, mamJ*, and *mamY* genes (Scheffel et al., 2006; Abreu et al., 2014; Toro-Nahuelpan et al., 2019). Among these, *mamJ* and *mamY* were not found in the available genomes of *Ca*. Etaproteobacteria, which indicates that other mechanisms of chain assembly and positioning have evolved in this group of MTB. By contrast, MamK that encodes an actin-like protein important for the chain organization is universally present in MTB, at least in those known to produce one or multiple chains (Komeili et al., 2006; Katzmann et al., 2010; Abreu et al., 2014). Therefore, the *mamK* gene was expected to be absent from the genome of UR-1, as no clear chains could be identified within the cells. However, the gene was found within magnetosome gene clusters of UR-1 and was even present as two copies.

The “unchained” magnetosomes in some magnetotactic cocci have often been observed to have a location proximal to the flagellar end of the cell rather than being distributed evenly in the cell body (Spring et al., 1995; Lin and Pan, 2009; Abreu et al., 2012; Zhang et al., 2012). One possibility is that the coordinated action of the two MamK proteins provides the specific organization and positioning of magnetosome clusters within the cell; however, which protein links the MamK filament and the magnetosome remains unclear. The MAI of UR-1 contains several genes encoding proteins with unknown functions, which may be candidates for this role. One of these genes, *maq1*, is located between *mamE* and *mamK* in “*Ca*. Magnetaquicoccaceae,” giving it a similar position to that of *mamJ* in *Magnetospirillum*. Our analysis shows that, in addition to *maq1*, the magnetosome gene clusters of “*Ca*. Magnetaquicoccaceae” contain a set of 11 genes that could be involved in magnetosome formation and magnetotaxis process. Since little could be predicted from the sequences of these genes, it is very tempting to speculate that other determinants of the clustered magnetosome organization might be found among them. Further research, preferably based on axenic cultures, is necessary to understand the genetic mechanisms of the biomineralization and magnetosome arrangement in magnetotactic cocci, including those forming clustered magnetosomes.

Although the position of UR-1 and WMHbinv6 on the trees based on the concatenated Mam proteins was congruent with the phylogenomic tree, an unusually high similarity rate of the magnetosome genes in comparison to the rest of the core genome was revealed. Since high conservation of magnetosome genes has never been observed, and it would contradict the bulk of data on the evolution of magnetosome genes, this finding suggests that magnetosome gene clusters could be transferred horizontally or from another closely related species.

Another interesting result of the magnetosome protein analyses is a non-congruent position of the branch consisting of “*Ca*. Magnetaquicoccaceae” magnetosome proteins when compared with the core protein tree. The phylogenetic analysis indicates that the branch consisting of “*Ca*. Magnetaquicoccaceae” magnetosome proteins was external to all other concatenates; also, the HCHbin5 concatenate did not cluster with those of closely related strains.

These results support the idea of an HGT of magnetosome genes within the order *Magnetococcales*. As suggested previously, vertical inheritance, followed by multiple independent losses of a magnetosome island, are likely to be the main driving forces for the evolution of magnetosome biomineralization genes. For some MTB (for example, for the genus *Magnetospirillum*), cases of possible independent HGT events have been demonstrated (Rioux et al., 2010; Komeili, 2012; Lefèvre et al., 2012a; Ji et al., 2017; Lin et al., 2018; Monteil et al., 2018). For example, *Mc. marinus* MC-1^T^ has a stable MAI (Schübbe et al., 2009), and biomineralization genes of both cultured *Magnetococcales* strains MC-1^T^ and IT-1 appeared as a result of the vertical inheritance (Morillo et al., 2014). Our results suggest that MAI in *Magnetococcales* strains could spread due to HGT, which occurred between closely related, as well as more distantly related groups within the order. We found a putative phage-related gene located directly downstream of *mamT* in the genome of UR-1 that had low identity with the phage capsid protein found in the genome of *Pseudoalteromonas neustonica* (WP_130470101; 33% identity; 23% coverage). Moreover, genes encoding putative phage-related proteins were also identified directly upstream of *feoB1*: WP_130471887, WP_130471886, and WP_130471885 contained phage tail, phage cell wall peptidase, and phage_BR0599 domains, respectively. Thus, the influence of bacteriophages could be a possible mechanism for horizontal transfer of magnetosome genes, at least in UR-1.

According to our analysis, HCHbin5 and UR-1 belong to the same genus, but due to putative HGT they had a noticeable difference in the structure of their MAI and had a low level of similarity of magnetosome genes. This may suggest that the magnetosomes of HCHbin5 are also arranged differently. Some studies have shown that magnetotactic cocci with different chain organizations were hybridized with the same specific FISH probe for the 16S rRNA gene (Spring et al., 1995; Cox et al., 2002; Lin and Pan, 2009). This led to the assumption that no strict correlation exists between the magnetosome organization within the cells and the phylogenetic position of magnetotactic cocci. Therefore, the appearance of magnetosomes is not a suitable phenotypic criterion for delimitation of taxonomic groups of MTB (Lin and Pan, 2009). Our analysis supports this assumption, due to the relative frequency of HGT revealed within the order *Magnetococcales*.

Several decades of studies on the diversity of MTB have led to the accumulation of a large amount of data on the 16S rRNA of “*Ca*. Etaproteobacteria.” The phylogenetic analysis of the 16S rRNA sequences indicated their significant diversity. The absence of cultivated freshwater representatives hampered classification (new sequences could be classified only at the order level); furthermore, the metabolic features of the representatives of the group remained elusive. Recently, several MAGs of freshwater *Magnetococcales* members have been reconstructed (Lin et al., 2018), and this has provided new opportunities for comparative genomic analyses and for establishing phylogenetic relationships within the order *Magnetococcales*.

Based on the results of our genomic analysis, we have proposed AAI thresholds for the separation of taxons of representatives of the class “*Ca*. Etaproteobacteria” at family and genus levels. Using the AAI values boundaries of 55–56% for the separation of families and 64–65% for the differentiation of close genera perfectly correlated with the branching of the phylogenomic tree. In accordance with these boundaries, five families within the order *Magnetococcales* were delineated. One of defined families has been named “*Ca*. Magnetaquicoccaceae” and includes the coccus UR-1 isolated from the Uda River. The comparative genomic analysis conducted in the current research sheds light on the potential metabolic abilities of the strain UR-1 as a representative of the candidate family “*Ca*. Magnetaquicoccaceae.” In addition, some metabolic diversity has been identified among the representatives of the proposed family (see the description below).

Overall, our analysis shows the fundamental differences in the genetic determinants for sulfur oxidation in UR-1 and the related MTB (with the exception of HA3dbin3), in comparison to the marine magnetic cocci MC-1^T^, MO-1, and IT-1. The marine forms appear to employ the reverse operating DsrAB in combination with the Sox pathway, whereas we predict that the freshwater forms use the reverse DsrAB, in combination with the APS reductase-mediated oxidation of sulfite.

Several key enzymes for poly-P synthesis were found in various marine and freshwater *Magnetococcales*, suggesting their important role in the phosphorus cycle (Lins and Farina, 1999; Schulz-Vogt et al., 2019). However, intriguingly, no genes for PPK1, the primary poly-P synthesis enzyme, were revealed in the available genomes. Although PPK2 is considered to have an activity that favors poly-P degradation, in the absence of PPK1, PPK2 can operate as synthesizing enzyme, albeit with less efficiency (Rao et al., 2009). The presence of multiple copies of *ppk2* gene in the sequenced genomes of *Magnetococcales* suggests a plausible scenario in which a novel poly-P synthesis-favoring type of PPK2 could evolve in this group of bacteria. To check this hypothesis, experiments with different PPK2 in the available cultivable representatives (MC-1, MO-1, or IT-1) should be conducted in the future.

In conclusion, the predicted metabolic features—the potential ability for chemolithoautotrophic growth with reduced sulfur compounds, nitrate respiration, the ability to assimilate nitrate and the expected relatively high tolerance to oxygen—may serve as useful guidelines for future attempts to isolate an axenic culture of UR-1 and related strains. These strains will be at the frontier of future research on this group of MTB.

### Description of Novel Candidate Family, Genus and Species, and Taxonomic Proposals

Genomic analysis of the UR-1 genome classified it as a novel candidate genus within a novel candidate family of the order *Magnetococcales*.

We propose the following Latin names for the novel candidate taxa:

*Candidatus* Magnetaquicoccaceae

Magnetaquicoccaceae (Mag.net.a.qui.coc.ca.ce'ae. N.L. masc. n. Magnetaquicoccus a *Candidatus* generic name; -*aceae* ending to denote a family; N.L. fem. pl. n. Magnetaquicoccaceae the (*Candidatus*) Magnetaquicoccus family).

Ca. “*Magnetaquicoccaceae”* is characterized by the potential ability for chemolithoautotrophic growth with the oxidation of reduced sulfur compounds through a reverse Dsr pathway (most enzymes from the Sox system are absent) and carbon assimilation by rTCA with the type II ATP:citrate lyase. The genes for nitrogen fixation are not universally found in the members of the family and might be limited to “*Ca*. Magnetaquicoccus inordinatus,” YD0425bin7 and WMHbin3. The genes for dissimilatory nitrate/nitrite/nitric and nitrous oxide reduction enzymes were found, but not for the entire pathway, indicating that the pathway might be truncated at different steps, depending on the species. The potential ability for assimilation of nitrate by NasA, which is, in general, absent from other *Magnetococcales*, was predicted for two members of the family: “*Ca*. Magnetaquicoccus inordinatus” and WMHbinv6.

*Candidatus* Magnetaquicoccus

Magnetaquicoccus (Mag.net.a.qui.coc'cus. Gr. n. *magnes*, -*etos* a magnet; N.L. pref. *magneto*- pertaining to a magnet; L. fem. n. *aqua* water; N.L. masc. n. *coccus* (from Gr. masc. n. *kokkos*), coccus; N.L. masc. n. *Magnetaquicoccus*, magnetic coccus from water).

*Candidatus* Magnetaquicoccus inordinatus

Magnetaquicoccus inordinatus (in.or.di.na' tus. N.L. masc. adj. *inordinatus* not arranged).

UR-1 cell has a coccoid morphology and represents magnetite magnetosomes not organized in chains and clustered in one side of the cell. Magnetosomes presents mean length of 77.4 nm (SD = 11.8 nm) and mean width of 46.2 nm (SD = 7.9 nm).

## Data Availability Statement

The datasets generated for this study can be found in the Genome project has been deposited in DDBJ/ENA/GenBank under the accession number RXIU00000000.

## Author Contributions

VK and DG conceived and designed experiments. VK collected the data. MK performed bioinformatics data processing. DG and MU carried out the phylogenetic and comparative genomic analyses. PL performed FISH-TEM analysis. VK, MD, and DG analyzed the data. VK and MD drafted the original manuscript. All authors read and approved the final manuscript.

### Conflict of Interest

The authors declare that the research was conducted in the absence of any commercial or financial relationships that could be construed as a potential conflict of interest.

## Supplementary Material

Supplementary File 1 Genomic reconstruction of cellular transport, oxidative stress defense strategies, chemotaxis and motility of UR-1.

Supplementary Figure S1 Control of FISH reaction using Uda 54-3 probe. **(A)** DIC image from *E. coli* culture. **(B)** *E. coli* cells stained with DAPI. **(C)** *E. coli* recognized by EUB probes. **(D)** *E. coli* cells not recognized by Uda 54-3 probe, indicating the specificity of this probe in the hybridization conditions described in this paper.

Supplementary Figure S2 Maximum-likelihood phylogenetic trees inferred from a comparison of concatenated magnetosome associated proteins MamABKLMOPST (2077 amino-acid sites) and MamEHI (885 amino-acid sites). Both trees reconstructed with evolutionary model LG+F+I+G4. The scale bar represents amino acid substitutions per site. Trees were rooted using two *Magnetospirillum* species, Zetaproteobacteria bacterium PCbin4 and Lambdaproteobacteria bacterium PCRbin3 as outgroup.

Supplementary Figure S3 Maximum-likelihood phylogenetic tree derived from the comparison of DsrAB sequences (1183 sequences) of sulfur-oxidizing and sulfate-reducing bacteria. Phylogenetic analysis was performed with a LG+F+I+G4 model based on 727 amino acid positions. The scale bar represents amino acid substitutions per site.

Supplementary Table S1 Physicochemical parameters of water at the sampling site.

Supplementary Table S2 Genome characteristics of “*Ca*. Magnetaquicoccus inordinatus” and other *Magnetococcales* strains used for comparison and phylogenetic reconstructions.

Supplementary Table S3 POCP values between strains of the order *Magnetococcales*. Black boarders separate the representatives belonging to different putative families; Red boarders separate putative genera.

Supplementary Table S4 ANI values between strains of the order *Magnetococcales*. Black boarders separate the representatives belonging to different putative families as was predicted by AAI analyses; Red boxes indicate same species; Orange boxes indicate putative same species.

Supplementary Table S5 dDDH values between strains of the order *Magnetococcales*. Black boarders separate the representatives belonging to different putative families as was predicted by AAI analyses; Red boxes indicate same species; Orange boxes indicate putative same species.

Supplementary Table S6 Results of analyses of putative magnetosome proteins of “*Ca*. Magnetaquicoccus inordinatus”.

Supplementary Table S7 Putatively horizontally transferred genes between “*Ca*. Magnetaquicoccus inordinatus UR-1” and Magnetococcales bacterium WMHbinv6. The percentage of genes similarity between UR-1 and WMHbinv6 is higher than between UR-1 and YD0425bin7 and between YD0425bin7 and WMHbinv6. This indicates the presence of horizontal genes transfer between UR-1 and WMHbinv6 and its absence between UR-1 and YD0425bin7 and between YD0425bin7 and WMHbinv6.

Supplementary Table S8 Distribution of the key genes for the major metabolic processes in the “*Ca*. Etaproteobacteria” members. “Y” (yes) indicates presence of a gene, “N” (no) indicates absence. Bold boarders separate the representatives belonging to different putative families according to the phylogenetic tree in Figure 3.
