# Supplementary material for: Genome-Based Metabolic Reconstruction of a Novel Uncultivated Freshwater Magnetotactic coccus “Ca. Magnetaquicoccus inordinatus” UR-1, and Proposal of a Candidate Family “Ca. Magnetaquicoccaceae”
Source: Front Microbiol. 2019 Oct 2;10:2290. doi: 10.3389/fmicb.2019.02290 (PMC6783814; doi:10.3389/fmicb.2019.02290)
Supplement: Supplementary File 1 — Genomic reconstruction of cellular transport, oxidative stress defense strategies, chemotaxis and motility of UR-1. [file Table_9.DOCX]

### Further metabolic features of UR-1 and related strains

Here we provide further details on metabolism of UR-1 and the group of related magnetotactic strains predicted on the basis of sequenced metagenomes, with focus on cellular transport, accessory genes in sulfur metabolism, and oxidative stress defense strategies.

**Transport**

The genome of UR-1 contains 130 genes encoding putative transporters and parts of the transporter systems, which is higher that the number of transporter related genes (115) predicted in *Magnetococcus marinus* MC-1^T^ (Schübbe et al., 2009). This number normalized to the genome size also exceeds that of MC-1^T^, having 31.4 per Mb in UR-1 vs. 24.5 per Mb in MC-1^T^. This is still low in comparison to many other bacteria with versatile heterotrophic metabolism, where the average value was calculated as 58.0 per Mb for 30 species by Schübbe et al. (2009), which might indicate the restricted heterotrophic abilities of UR-1 similar to the cultivated magnetotactic cocci.

Putative ATP-binding cassette (ABC) transport systems are represented in the genome of UR-1 by 50 genes. ABC transporters utilize the energy of ATP hydrolysis to transfer substrate, which is usually relatively specific for a particular transporter, across the membrane against a concentration gradient (Higgins, 1992). Full sets of genes encoding ABC transporters for several essential microelements were identified in the genome of UR-1: molybdate transport system ModABC, zinc transporter ZnuABC and iron III transporter AfuABC. Interestingly, AfuABC is absent from genomes of MC-1^T^ and MO-1, where in spite of the need for iron ions for magnetosome biosynthesis, the iron transporters are scarce (Schübbe et al., 2009; see also this research, Supplementary Table S8). These three transporters were also identified in the closely related WMHbinv6, although AfuABC could not be found in YD0425bin7, another member of the same group. Unlike in MC-1^T^, the predicted ABC cobalt/nickel transporter operon Cbi/NikMNQO is incomplete in UR-1, lacking all the parts with the exception of the single ATP-binding protein CbiO. Although nickel (Ni) and cobalt (Co) are utilized at very low levels in comparison to other trace elements, they are essential components of several metalloenzymes (Ni) and vitamins (Co) (Mulrooney and Hausinger, 2003), (Banerjee and Ragsdale, 2003). For uptake of Co and Ni, which present in the natural environments at very low amounts, most bacteria relay on high-affinity transport systems, with the most wide-spread system Cbi/NikMNQO, and several secondary transporters (Zhang et al., 2009). In the closely related WMHbinv6 the transporter Cbi/NikMNQO was also not found (except CbiO), as well as in several more distantly related freshwater magnetotactic cocci HCHbin5, ER1bin7 and WMHbin3, suggesting that these species as well as UR-1 may relay on secondary transporters for Co and Ni uptake. In the genome of UR-1 three putative MgtE superfamily and one CorA/ZntB-like transporters were predicted, which can potentially adopt the role of Ni and Co uptake. MgtE and CorA are the most studied ubiquitously distributed primary transporters for Mg^2+^ ions, MgtE also regulates Mg^2+^ homeostasis (Hattori et al., 2009). It has been shown that both systems are capable of Co2+ uptake, however with lower affinity, than Mg2+ (Hmiel et al., 1986). The redundancy in the number of MgtE-like transporters, which is also intrinsic to the other closely related magnetotactic cocci WMHbinv6, YD0425bin7 and HCHbin5, may also suggest that some of them specialized on the Co or/and Ni uptake in addition or instead if Mg^2+^. It is also possible that other novel Co/Ni transporters are still to be identified in these bacteria.

Similar to MC-1^T^, genome of UR-1 comprises the entire set of ABC transporters for branched-chain amino acids and urea. In addition, UR-1 possesses glutamate/aspartate transporter GltKLI, which was not found in the genomes of the other freshwater *Magnetococcales* from metagenomic samples, with a few exceptions (WMHbinv6, YD0425bin7 and WMHbin3). High-affinity ABC transport systems for phosphate encoded in the *pstSCAB-phoU* operon and for the nitrate/nitrite transporter NrtABC were also found in UR-1. The presence of NrtABC correlates with the predicted ability of UR-1 for respiratory nitrate and nitrite reduction through Nar and Nir complexes respectively. No putative high-affinity ABC transporters for sulfate were predicted in the genome of UR-1. Instead, similar to the cultivated strains MC-1^T^ and MO-1, it putatively relies on the inorganic anion transporters of the sulfate permease (SulP) family.

**Putative accessory genes in sulfur metabolism**

In addition to major operons putatively involved in the metabolism of reduced sulfur compounds, an operon comprising genes similar to *hdr* was also found in the genome of UR-1 and several other freshwater *Magnetococcales*. *Hdrs* encode heterosulfide-reductases, enzymes present in methanogenic archea, which catalyze the reduction of the heterosulfide, formed in the last step of methanogenesis (Wagner et al., 2017). It has been demonstrated that in *Hyphomicrobium denitrificans* a heterosulfide-reductase Hdr-like system acts as a sulfur-oxidizing entity in the cytoplasm and is a key player in oxidation of thiosulfate to sulfate and degradation of dimethylsulfide (DMS) (Koch and Dahl, 2018). The organization of the *hdr*-like genes in the UR-1 genome differs from that in the well-studied sulfur-oxidizing bacteria, e.g. *Acidithiobacillus caldus*, *Thioalkalivibrio* spp*.*, or *H. denitrificans*, where the *hdr* genes were found to have the same order and content: *hdrC1B1A-hyp-hdrC2B2* (Dahl 2015*;* Koch and Dahl 2018)*.* In UR-1 the putative *hdrA1A2CB* operon includes only four genes. The domain prediction by InterProScan (Quevillon et al., 2005) reveals the delta subunit of methyl-viologen-reducing hydrogenase FlpD localized to the C-terminus of the putative HdrA2, in addition to the 4Fe-4S sununit A (HdrA) domain, similar to organization of the HdrA-like protein in a methanogenic sulfate-reducing archeon Archaeoglobus fulgidus (Hocking et al., 2014). Contradictory, the minimalistic organization of the operon and presence of a hydrogenase subunit in the putative protein suggests that the operon encodes rather the proteins functionally similar the other Hdr-like proteins - the QmoABC complex of sulfate-reducing and some sulfur oxidizing bacteria. QmoABC is involved in the donation of electrons to adenylylsulfate reductase AprAB, linking the quinone pool to sulfate reduction or APS oxidation (Ramos et al., 2012). On the contrary, QmoABC should also include a cytochrome b containing transmembrane domain, which has not been found among Hdr-like proteins of UR-1. Therefore, it is difficult to predict with confidence, if this set of the *hdr*-like genes of UR-1 acts as Hdr sulfur reductase or as Qmo complex. It is also worth noting that Hdr-like pathway in sulfur-oxidizing is viewed to be a functional substitution for the Dsr pathway, as the genes for the both of them almost never occur in the same genome, suggesting that using either one of the pathways is the rule rather than the exception (Koch and Dahl, 2018). As a complete set of genes for the oxidizing type of Dsr enzymes is present in UR-1, it supports the hypothesis of the alternative function of *hdr*-like genes in this organism.

**Chemotaxis and motility**

As any other MTB, UR-1 and related freshwater magnetotactic strains inhabit anoxic and oxic-anoxic interface zones (OAI) in the aquatic sediments, where motility in cooperation with the passive alignment in the magnetic field (i.e., magneto-aerotaxis or magnetotaxis) plays a crucial role in the adaptation of these organisms to the stratified and ever-changing environment. It has been demonstrated previously that marine magnetotactic cocci MC-1^T^ and MO-1 swim by means of a flagellar apparatus consisting of two sheathed flagellar bundles, which have complex, highly ordered spatial organization (Zhang et al. 2012; Ruan et al. 2012). Such a robust locomotion mechanism is deemed to be specific to marine magnetotactic ovoid-shaped bacteria, serving as ingenious adaptation to their habitat (Ji et al. 2016; Zhang et al. 2012). This also appears to correlate with the unusually high number of the flagellin *fliC* genes found in the genomes of MC-1^T^ and MO-1, i.e. 15 and 14 respectively. It is noteworthy, that the presence of more than 7 *fliC* genes is intrinsic to magnetotactic cocci, and such a number has never been found in other groups (Zhang et al. 2012). Due to the absence of cultivated representatives, little is known about the flagella organization in freshwater *Magnetococcales*. The TEM micrographs obtained for UR-1 in the current research do not reveal flagella at sufficient resolution. However, our analysis of the genomic data allows several predictions. In consistence with the previous observation on the unusually high number of the flagellin genes in magnetic ovoid-shaped bacteria, genome of UR-1 contains in total 10 *fliC* genes that could be an indication of complexity of the flagella apparatus in this organism. The flagella assembly gene clusters comprise the full set of genes for type III flagellar export pathway, assembly regulators and chaperones, flagellar structural genes for the basal body, hook and filament, as well as motor and motor switch proteins (Table S9).

Our analysis revealed 76 genes encoding the proteins homologous to the core chemotaxis two-component system (CheA, CheY, CheW) as well as accessory players participating in signal recognition and transduction (methyl-accepting chemotaxis proteins, MCP; CheD), adaptation (CheR, CheB, CheV) and signal removal (phosphatases CheC, CheX) in the genome of UR-1 (Szurmant and Ordal, 2004). Most of the central components of chemotaxis are represented by multiple genes (the number of genes in the genome is indicated in parenthesis; see also Table S10), e.g. MCP (30), sensor kinase CheA (7), the coupling protein CheW (8), key response regulator CheY (8), adaptation methyl transferase CheR (8), methyl esterase CheB (6), phosphatases CheC and CheX (3 each). Genome of UR-1 is also rich in MCP receptors (30), similar to other *Magnetococcales* strains, e.g. 32 in MC-1^T^ (Schübbe et al., 2009). Localization of some genes at the edges of the contigs does not allow determining the exact number and size of the chemotaxis gene clusters as some of them might be adjacent one to another. In general, the redundancy in the key *che* genes indicates the potential complexity of chemotactic behavior in UR-1 and its importance for the adaptation of the organism to the natural environment.

Table S9. Genes encoding flagellar apparatus assembly in UR-1

| **Protein ID** | **Gene** | **Common name and function of the product** |
| --- | --- | --- |
| **Basal body** | | |
| WP_130470626 | *fliF* | Flagellar M-ring protein FliF |
| WP_130472139 | *flgI* | Flagellar P-ring protein precursor FlgI |
| WP_130472137 | *flgA* | Flagella basal body P-ring formation protein FlgA |
| WP_130472138 | *flgH* | Flagellar L-ring protein precursor FlgH |
| WP_130470629 | *flgB* | Basal-body rod protein FlgB |
| WP_130470628 | *flgC* | Basal-body rod protein FlgC |
| WP_130470454 | *flgD* | Basal-body rod modification protein FlgD |
| WP_130472927 | *flgG* | Basal-body rod protein FlgG |
| WP_130472136 | *flgG* | Basal-body rod protein FlgG |
| WP_130470455 | *flgE* | Hook protein FlgE |
| **Flagellar motor/motor switch** | | |
| WP_130472046 | *motA* | Stator protein, exerts torque against rotor/switch |
| WP_130472045 | *motB* | Stator protein, converts proton energy into torque |
| WP_130472549 | *fliG* | Flagellar motor switch protein FliG |
| WP_130470625 | *fliG* | Flagellar motor switch protein FliG |
| WP_130470457 | *fliM* | Flagellar motor switch protein FliM, target for CheY-P binding |
| WP_130470464 | *fliNY* | Flagellar motor switch protein FliN/FliY |
| **Rod, hook and filament** | | |
| WP_130470627 | *fliE* | Hook-basal body complex protein FliE |
| WP_130471141 | *flgK* | Hook-associated protein 1 FlgK |
| WP_130471148 | *fliD* | Hook-associated protein 2 FliD |
| WP_130470456 | *fliL* | FliL protein |
| WP_130472843 | *fliC* | Filament protein, flagellin |
| WP_130472835 | *fliC* | Filament protein, flagellin |
| WP_130472683 | *fliC* | Filament protein, flagellin |
| WP_130472461 | *fliC* | Filament protein, flagellin |
| WP_130472477 | *fliC* | Filament protein, flagellin |
| WP_130472365 | *fliC* | Filament protein, flagellin |
| WP_130472330 | *fliC* | Filament protein, flagellin |
| WP_130471144 | *fliC* | Filament protein, flagellin |
| WP_130471146 | *fliC* | Filament protein, flagellin |
| WP_130470830 | *fliC* | Filament protein, flagellin |
| WP_130472846 | *flaG* | flagellar protein FlaG |
| **Type III flagellar secretion system** | | |
| WP_130470458 | *fliO* | Export component |
| WP_130470623 | *fliI* | Flagellum-specific ATP-synthase |
| WP_130470624 | *fliH* | Negative regulator of FliI |
| WP_130470465 | *fliP* | Export component |
| WP_130472367 | *fliQ* | Export component |
| WP_130472368 | *fliR* | Export component |
| WP_130471704 | *flhA* | Export component |
| WP_130472369 | *flhB* | Export component, substrate specificity switch |
| **Regulatory proteins** | | |
| WP_130471700 | *fliA* | RNA polymerase sigma factor for flagellar operon FliA |
| WP_130472141 | *flgM* | Negative regulator of flagellin synthesis FlgM |
| WP_130471698 | *flhF* | Flagellar biosynthesis protein FlhF |
| WP_130471699 | *flhG* | flagellar biosynthesis protein FlhG |
| WP_130470453 | *fliK* | Flagellar hook-length control protein FliK |
| **Flagellar assembly chaperons** | | |
| WP_130471150 | *fliS* | Flagellar export chaperone FliS |
| WP_130471149 | *fliS* | Flagellar export chaperone FliS |
| WP_130471145 | *fliW* | Flagellar assembly factor FliW |
| WP_130471143 | *fliW* | Flagellar assembly factor FliW |

Table S10. Chemotaxis genes found in the genome of UR-1

| **Protein ID** | **Gene** | **Common name and function of the product** |
| --- | --- | --- |
| **Signal recognition and transduction** | | |
| WP_130472755 | *cheD* | chemotaxis glutamine deaminase CheD |
| WP_130470671 | *cheD* | chemotaxis glutamine deaminase CheD |
| **Methyl-accepting chemotaxis protein (MCP)** | | |
| WP_130472666 | *mcp* | methyl-accepting chemotaxis protein |
| WP_130471638 | *mcp* | methyl-accepting chemotaxis protein |
| WP_130471037 | *mcp* | methyl-accepting chemotaxis protein |
| WP_130471039 | *mcp* | methyl-accepting chemotaxis protein |
| WP_130469983 | *mcp* | methyl-accepting chemotaxis protein |
| WP_130472813 | *mcp* | methyl-accepting chemotaxis protein |
| WP_130472629 | *mcp* | methyl-accepting chemotaxis protein |
| WP_130472696 | *mcp* | methyl-accepting chemotaxis protein |
| WP_130472659 | *mcp* | methyl-accepting chemotaxis protein |
| WP_130472336 | *mcp* | methyl-accepting chemotaxis protein |
| WP_130472219 | *mcp* | methyl-accepting chemotaxis protein |
| WP_130472037 | *mcp* | methyl-accepting chemotaxis protein |
| WP_130471981 | *mcp* | methyl-accepting chemotaxis protein |
| WP_130471975 | *mcp* | methyl-accepting chemotaxis protein |
| WP_130471979 | *mcp* | methyl-accepting chemotaxis protein |
| WP_130471832 | *mcp* | methyl-accepting chemotaxis protein |
| WP_130471709 | *mcp* | methyl-accepting chemotaxis protein |
| WP_130471728 | *mcp* | methyl-accepting chemotaxis protein |
| WP_130471736 | *mcp* | methyl-accepting chemotaxis protein |
| WP_130471598 | *mcp* | methyl-accepting chemotaxis protein |
| WP_130471407 | *mcp* | methyl-accepting chemotaxis protein |
| WP_130471403 | *mcp* | methyl-accepting chemotaxis protein |
| WP_130471404 | *mcp* | methyl-accepting chemotaxis protein |
| WP_130471189 | *mcp* | methyl-accepting chemotaxis protein |
| WP_130471168 | *mcp* | methyl-accepting chemotaxis protein |
| WP_130471200 | *mcp* | methyl-accepting chemotaxis protein |
| WP_130470779 | *mcp* | methyl-accepting chemotaxis protein |
| WP_130470367 | *mcp* | methyl-accepting chemotaxis protein |
| WP_130470043 | *mcp* | methyl-accepting chemotaxis protein |
| WP_130469984 | *mcp* | methyl-accepting chemotaxis protein |
| **Central components of chemotaxis two-component system** | | |
| WP_130471424 | *cheA* | two-component system, sensor histidine kinase CheA |
| WP_130471169 | *cheA* | two-component system, sensor histidine kinase CheA |
| WP_130472907 | *cheA* | two-component system, sensor histidine kinase CheA |
| WP_130472656 | *cheA* | two-component system, sensor histidine kinase CheA |
| WP_130471399 | *cheA* | two-component system, sensor histidine kinase CheA |
| WP_130470665 | *cheA* | two-component system, sensor histidine kinase CheA |
| WP_130470044 | *cheA* | two-component system, sensor histidine kinase CheA |
| WP_130472600 | *cheW* | coupling chemotaxis protein CheW |
| WP_130471735 | *cheW* | coupling chemotaxis protein CheW |
| WP_130471429 | *cheW* | coupling chemotaxis protein CheW |
| WP_130471428 | *cheW* | coupling chemotaxis protein CheW |
| WP_130471402 | *cheW* | coupling chemotaxis protein CheW |
| WP_130471175 | *cheW* | coupling chemotaxis protein CheW |
| WP_130471034 | *cheW* | coupling chemotaxis protein CheW |
| WP_130471036 | *cheW* | coupling chemotaxis protein CheW |
| WP_130472597 | *cheY* | two-component system, primary response regulator CheY |
| WP_130472196 | *cheY* | two-component system, primary response regulator CheY |
| WP_130472193 | *cheY* | two-component system, primary response regulator CheY |
| WP_130471835 | *cheY* | two-component system, primary response regulator CheY |
| WP_130471400 | *cheY* | two-component system, primary response regulator CheY |
| WP_130470986 | *cheY* | two-component system, primary response regulator CheY |
| WP_130470098 | *cheY* | two-component system, primary response regulator CheY |
| WP_130469998 | *cheY* | two-component system, primary response regulator CheY |
| **Chemotaxis adaptation** | | |
| WP_130472192 | *cheR* | chemotaxis methyltransferase CheR |
| WP_130472626 | *cheR* | chemotaxis methyltransferase CheR |
| WP_130472598 | *cheR* | chemotaxis methyltransferase CheR |
| WP_130472200 | *cheR* | chemotaxis methyltransferase CheR |
| WP_130471167 | *cheR* | chemotaxis methyltransferase CheR |
| WP_130471035 | *cheR* | chemotaxis methyltransferase CheR |
| WP_130471005 | *cheR* | chemotaxis methyltransferase CheR |
| WP_130470531 | *cheR* | chemotaxis methyltransferase CheR |
| WP_130471398 | *cheB* | chemotaxis methylesterase CheB |
| WP_130472599 | *cheB* | chemotaxis methylesterase CheB |
| WP_130472228 | *cheB* | chemotaxis methylesterase CheB |
| WP_130471430 | *cheB* | chemotaxis methylesterase CheB |
| WP_130471004 | *cheB* | chemotaxis methylesterase CheB |
| WP_130472955 | *cheBR* | Chemotaxis methyltransferase/methylesterase CheB/CheR fusion protein |
| WP_130471405 | *cheV* | two-component system, coupling and adaptation CheV, fusion of CheW and CheY |
| WP_130470739 | *cheV* | two-component system, coupling and adaptation CheV, fusion of CheW and CheY |
| **Phosphate removal** | | |
| WP_130472332 | *cheX* | chemotaxis phosphatase CheX |
| WP_130471606 | *cheX* | chemotaxis phosphatase CheX |
| WP_130470958 | *cheX* | chemotaxis phosphatase CheX |
| WP_130470667 | *cheC* | chemotaxis phosphatase CheC |
| WP_130470670 | *cheC* | chemotaxis phosphatase CheC |

**Oxygen stress defense strategies**

Since MTB inhabit suboxic and anoxic habitats in the aquatic sediments, they are in general less exposed to the damage by reactive oxygen species (ROS) in comparison to aerobic bacteria. In consistence to this, they are characterized by relatively scarce number of genes involved in the oxygen stress defense. The activity tests showed that *Magnetospirillum* spp. are usually lacking the catalase activity, but some possess cytochrome *c* peroxidase, with the exception of *Magnetospirillum gryphiswaldense* MSR-1^T^, which demonstrates both activities and is apparently more aerotolerant than other magnetospirilla (Dziuba et al., 2016; Schleifer et al., 1991). Some other complex systems to cope with intracellular oxidative stress were found in the genomes of MTB from family *Rhodospirillaceae* (Wang et al., 2019). The genes participating in the detoxification of ROS in MC-1^T^ are limited to cytochrome *c* peroxidases and alkyl hydroperoxide reductase, whereas superoxide dismutase and catalase were not found (Schübbe et al., 2009). Unlike MC-1^T^, two other cultivated marine magnetotactic cocci, MO-1 and IT-1, possess genes for superoxide dismutase, which putatively adds the additional level of protection from ROS for these species. In contrast to almost all analyzed magnetotactic strains (with the exception of IT-1) UR-1 and the closely related metagenome WMHbinv6 have both superoxide dismutase and catalase I, in addition to various number of cytochrome *c* peroxidases (2 genes in UR-1, 1 in WMHbinv6), thiol peroxidase and alkyl peroxidase (Supplementary Table S8). This might indicate the higher tolerance to the oxidative stress in these species in comparison to other *Magnetococcales*. The other analyzed metagenomes of the freshwater magnetotactic strains, even the closely related to UR-1 YD0425bin7 and HCHbin5, seem to encode in general fewer genes for ROS detoxification. Several metagenomes reveal even more extreme limitation in the known oxidative stress defense genes, e.g. in ER1bin7, HA3dbin1 and Haa3bin1. This might be caused by the incomplete state of the genomes, or alternatively suggests that these species may be more selective in the oxygen level preferences while navigating in their native habitats.

**References**

Banerjee, R., and Ragsdale, S. W. (2003). The many faces of vitamin B12. 209–247. doi:10.1146/annurev.biochem.72.121801.161828.

Dahl, C. (2015). Cytoplasmic sulfur trafficking in sulfur-oxidizing prokaryotes. *IUBMB Life* 67, 268–274. doi:10.1002/iub.1371.

Dziuba, M., Koziaeva, V., Grouzdev, D., Burganskaya, E., Baslerov, R., Kolganova, T., et al. (2016). Magnetospirillum caucaseum sp. Nov., magnetospirillum marisnigri sp. Nov. and magnetospirillum moscoviense sp. Nov., freshwater magnetotactic bacteria isolated from three distinct geographical locations in European Russia. *Int. J. Syst. Evol. Microbiol.* 66. doi:10.1099/ijsem.0.000994.

Hattori, M., Iwase, N., Furuya, N., Tanaka, Y., Tsukazaki, T., Ishitani, R., et al. (2009). Mg 2-dependent gating of bacterial MgtE channel underlies Mg 2 homeostasis. *EMBO J.* 28, 3602–3612. doi:10.1038/emboj.2009.288.

Higgins, C. (1992). ABC Transporters: From Mircoorganisms to Man. *Annu. Rev. Cell Dev. Biol.* 8, 67–113. doi:10.1146/annurev.cellbio.8.1.67.

Hmiel, S. P., Snavely, M. D., Miller, C. G., and Maguire, M. E. (1986). Magnesium Transport in Salmonella typhimurium: Characterization of Magnesium Influx and Cloning of a Transport Gene. *J. Bacteriol.* 168, 1444–1450.

Hocking, W. P., Stokke, R., Roalkvam, I., and Steen, I. H. (2014). Identification of key components in the energy metabolism of the hyperthermophilic sulfate-reducing archaeon Archaeoglobus fulgidus by transcriptome analyses. *Front. Microbiol.* 5, 1–20. doi:10.3389/fmicb.2014.00095.

Ji, B., Zhang, S., Zhang, W., Rouy, Z., Alberto, F., Zhang, L., et al. (2017). The chimeric nature of the genomes of marine magnetotactic coccoid-ovoid bacteria defines a novel group of Proteobacteria. *Environ. Microbiol.* 19, 1103–1119. doi:10.1111/1462-2920.13637.

Koch, T., and Dahl, C. (2018). A novel bacterial sulfur oxidation pathway provides a new link between the cycles of organic and inorganic sulfur compounds. *ISME J.* 12, 2479–2491. doi:10.1038/s41396-018-0209-7.

Mulrooney, S. B., and Hausinger, R. P. (2003). Nickel uptake and utilization by microorganisms. *FEMS Microbiol. Rev.* 27, 239–261. doi:10.1016/S0168-6445(03)00042-1.

Quevillon, E., Silventoinen, V., Pillai, S., Harte, N., Mulder, N., Apweiler, R., et al. (2005). InterProScan: protein domains identifier. *Nucleic Acids Res.* 33, W116–W120. doi:10.1093/nar/gki442.

Ramos, A. R., Keller, K. L., Wall, J. D., and Cardoso Pereira, I. A. (2012). The membrane qmoABC complex interacts directly with the dissimilatory adenosine 5′-phosphosulfate reductase in sulfate reducing bacteria. *Front. Microbiol.* 3, 1–10. doi:10.3389/fmicb.2012.00137.

Ruan, J., Kato, T., Santini, C.-L., Miyata, T., Kawamoto, A., Zhang, W.-J., et al. (2012). Architecture of a flagellar apparatus in the fast-swimming magnetotactic bacterium MO-1. *Proc. Natl. Acad. Sci.* 109, 20643–20648. doi:10.1073/pnas.1215274109.

Schleifer, K. H., Schüler, D., Spring, S., Weizenegger, M., Amann, R., Ludwig, W., et al. (1991). The Genus Magnetospirillum gen. nov. Description of Magnetospirillum gryphiswaldense sp. nov. and Transfer of Aquaspirillum magnetotacticum to Magnetospirillum magnetotacticum comb. nov. *Syst. Appl. Microbiol.* 14, 379–385. doi:10.1016/S0723-2020(11)80313-9.

Schübbe, S., Williams, T. J., Xie, G., Kiss, H. E., Brettin, T. S., Martinez, D., et al. (2009). Complete Genome Sequence of the Chemolithoautotrophic Marine Magnetotactic Coccus Strain MC-1. 75, 4835–4852. doi:10.1128/AEM.02874-08.

Szurmant, H., and Ordal, G. W. (2004). Diversity in Chemotaxis Mechanisms among the Bacteria and Archaea. *Microbiol. Mol. Biol. Rev.* 68, 301–319. doi:10.1128/mmbr.68.2.301-319.2004.

Wagner, T., Koch, J., Ermler, U., and Shima, S. (2017). Methanogenic heterodisulfide reductase (HdrABC-MvhAGD) uses two noncubane [4Fe-4S] clusters for reduction. *Science (80-. ).* 357, 699–703. doi:10.1126/science.aan0425.

Wang, Y., Casaburi, G., Lin, W., Li, Y., Wang, F., and Pan, Y. (2019). Genomic evidence of the illumination response mechanism and evolutionary history of magnetotactic bacteria within the Rhodospirillaceae family. *BMC Genomics*. doi:10.1186/s12864-019-5751-9.

Zhang, W. J., Santini, C. L., Bernadac, A., Ruan, J., Zhang, S. Da, Kato, T., et al. (2012). Complex spatial organization and flagellin composition of flagellar propeller from marine magnetotactic ovoid strain MO-1. *J. Mol. Biol.* 416, 558–570. doi:10.1016/j.jmb.2011.12.065.

Zhang, Y., Rodionov, D. A., Gelfand, M. S., and Gladyshev, V. N. (2009). Comparative genomic analyses of nickel, cobalt and vitamin B12 utilization. *BMC Genomics* 10. doi:10.1186/1471-2164-10-78.
