# Supplementary figures and images for: Genome-Based Metabolic Reconstruction of a Novel Uncultivated Freshwater Magnetotactic coccus “Ca. Magnetaquicoccus inordinatus” UR-1, and Proposal of a Candidate Family “Ca. Magnetaquicoccaceae”
Source: Front Microbiol. 2019 Oct 2;10:2290. doi: 10.3389/fmicb.2019.02290 (PMC6783814; doi:10.3389/fmicb.2019.02290)

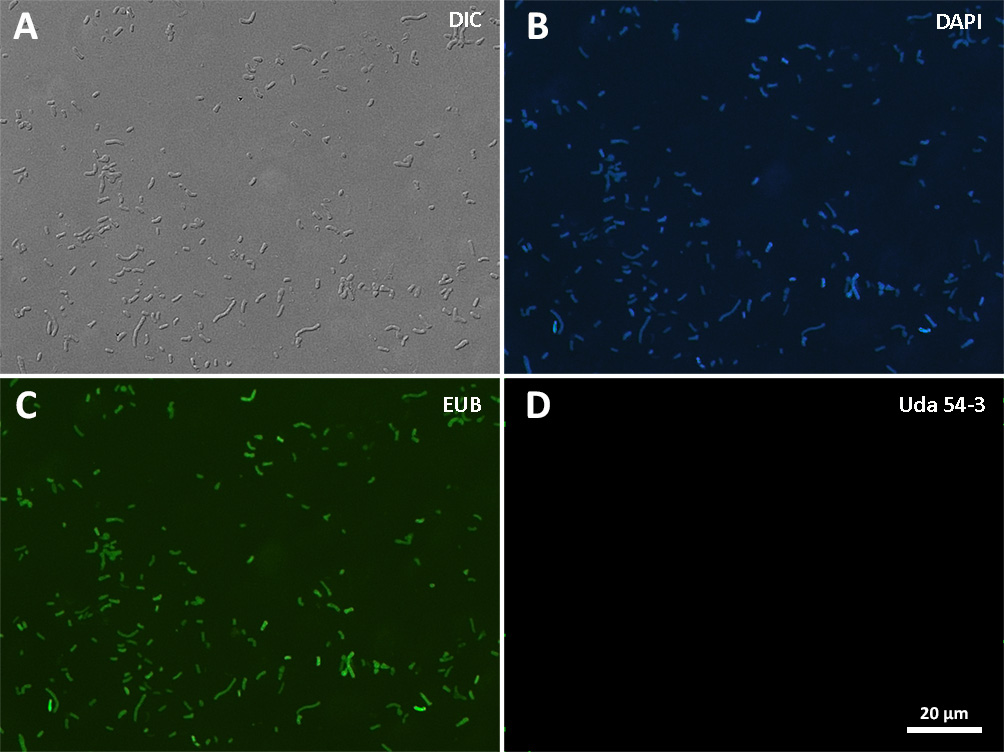

Supplement: Supplementary Figure S1 — Control of FISH reaction using Uda 54-3 probe. (A) DIC image from E. coli culture. (B) E. coli cells stained with DAPI. (C) E. coli recognized by EUB probes. (D) E. coli cells not recognized by Uda 54-3 probe, indicating the specificity of this probe in the hybridization conditions described in this paper. [file Image_1.JPEG]

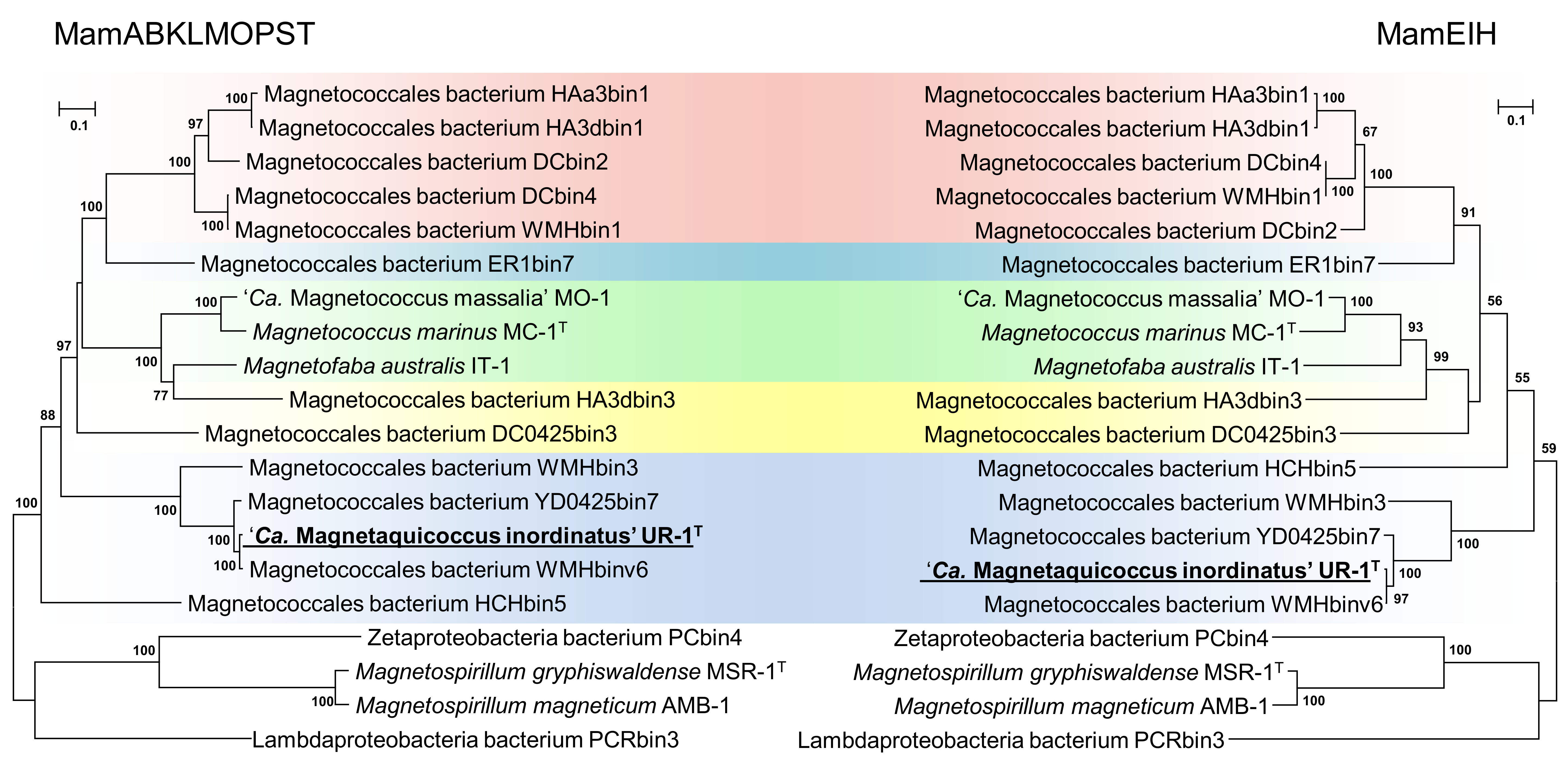

Supplement: Supplementary Figure S2 — Maximum-likelihood phylogenetic trees inferred from a comparison of concatenated magnetosome associated proteins MamABKLMOPST (2077 amino-acid sites) and MamEHI (885 amino-acid sites). Both trees reconstructed with evolutionary model LG+F+I+G4. The scale bar represents amino acid substitutions per site. Trees were rooted using two Magnetospirillum species, Zetaproteobacteria bacterium PCbin4 and Lambdaproteobacteria bacterium PCRbin3 as outgroup. [file Image_2.TIFF]

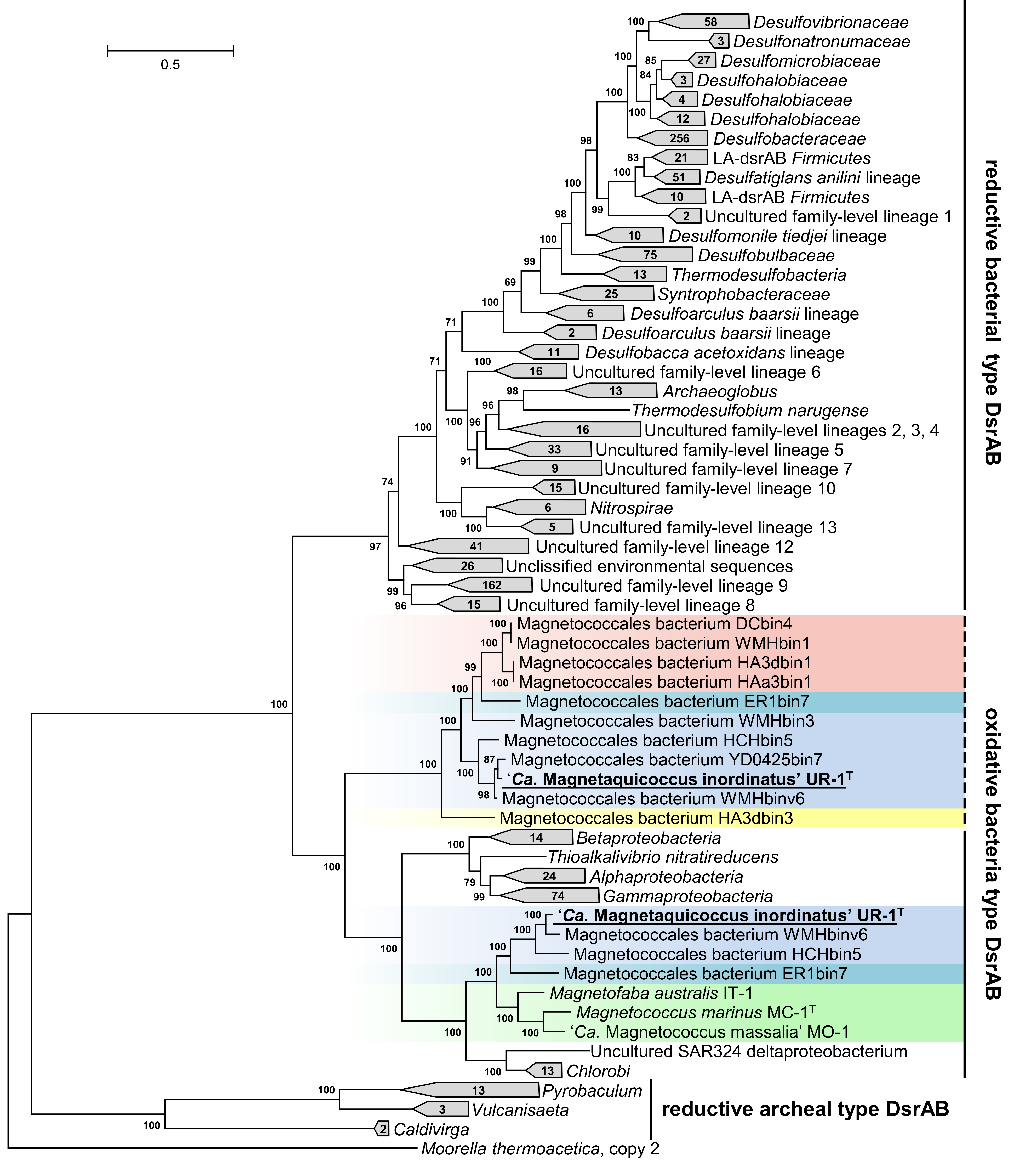

Supplement: Supplementary Figure S3 — Maximum-likelihood phylogenetic tree derived from the comparison of DsrAB sequences (1183 sequences) of sulfur-oxidizing and sulfate-reducing bacteria. Phylogenetic analysis was performed with a LG+F+I+G4 model based on 727 amino acid positions. The scale bar represents amino acid substitutions per site. [file Image_3.TIFF]
