# Supplementary material for: Genome-Based Metabolic Reconstruction of a Novel Uncultivated Freshwater Magnetotactic coccus “Ca. Magnetaquicoccus inordinatus” UR-1, and Proposal of a Candidate Family “Ca. Magnetaquicoccaceae”
Source: Front Microbiol. 2019 Oct 2;10:2290. doi: 10.3389/fmicb.2019.02290 (PMC6783814; doi:10.3389/fmicb.2019.02290)
Supplement: Supplementary Table S8 — Distribution of the key genes for the major metabolic processes in the “Ca. Etaproteobacteria” members. “Y” (yes) indicates presence of a gene, “N” (no) indicates absence. Bold boarders separate the representatives belonging to different putative families according to the phylogenetic tree in Figure 3. [file Table_8.DOCX]

Supplementary Table S8. Distribution of the key genes for the major metabolic processes in the *Magnetococcales* strains. ‘Y’ (yes) indicates presence of a gene, ‘N’ (no) indicates absence. Bold boarders separate the representatives belonging to different putative families according to the phylogenetic tree in Figure 3.

| **Gene symbol** | **Gene product** | | ***‘Ca.* Magnetaquicoccaceae’** | | | | | Family WMHbin1 | | | | | ER1bin7 | | Family DC0425bin3 | | *Magnetococcaceae* | | |
| --- | --- | --- | --- | --- | --- | --- | --- | --- | --- | --- | --- | --- | --- | --- | --- | --- | --- | --- | --- |
|  |  |  | **UR-1** | WMHbinv6 | YD0425bin7 | HCHbin5 | WMHbin3 | WMHbin1 | DCbin2 | DCbin4 | HA3dbin1 | Haa3bin1 | | ER1bin7 | DC0425bin3 | HA3dbin3 | MC-1 | MO-1 | IT-1 |
| **Carbon** | | | | | | | | | | | | | | | | | | | |
| **CO fixation through rTCA** | | | | | | | | | | | | | | | | | | | |
| *aclA* | ATP citrate lyase sub. A | | **Y** | Y | Y | Y | Y | N | N | N | N | N | | Y | Y | Y | Y | Y | Y |
| *aclB* | ATP citrate lyase sub. B | | **Y** | Y | Y | Y | Y | N | N | N | N | N | | Y | Y | Y | Y | Y | Y |
| *porA* | pyruvate: ferredoxin oxidoreductase Fe-S sub. | | **Y** | Y | Y | Y | Y | Y | Y | Y | Y | Y | | Y | Y | Y | Y | Y | Y |
| *porB* | pyruvate: ferredoxin oxidoreductase beta subunit | | **Y** | Y | Y | Y | Y | Y | Y | Y | Y | Y | | Y | Y | Y | Y | Y | Y |
| *korA* | 2-oxoglutarate:ferredoxin oxidoreductase, alpha | | **Y (x2)** | Y (x2) | Y (x2) | Y | Y (x2) | Y (x2) | Y | Y (x2) | Y (x2) | Y (x2) | | Y | Y | Y | Y | Y (x2) | Y |
| *korB* | 2-oxoglutarate:ferredoxin oxidoreductase, beta | | **Y (x2)** | Y (x2) | Y (x2) | Y | Y (x2) | Y (x2) | Y | Y (x2) | Y (x2) | Y (x2) | | Y | Y | Y | Y | Y (x2) | Y |
| **Oxidative TCA** | | | | | | | | | | | | | | | | | | | |
| *gltA* | citric acid syntase | | **Y** | Y | Y | Y | Y | Y | Y | Y | Y | Y (x2) | | Y | Y | Y (x2) | Y | Y | Y |
| *acs* | acetyl-CoA synthetase | | **Y** | Y | Y | Y | Y | Y | Y | Y | Y | Y | | Y | Y | Y | Y | Y | Y |
| *pck* | phosphoenol- pyruvate carboxykinase | | **Y** | Y | Y | Y | Y | N | N | N | N | N | | Y | N | N | Y | Y | Y |
| **Nitrogen** | | | | | | | | | | | | | | | | | | | |
| **Nitrogen fixation** | | | | | | | | | | | | | | | | | | | |
| *nifZ* | nitrogen fixation protein NifZ | | **N** | N | N | N | N | N | N | N | Y | Y | | N | N | N | Y | Y | Y |
| *nifV* | homocitrate synthase NifV | | **Y** | N | Y | N | Y | Y | N | Y | Y | Y | | N | Y | Y | Y | Y | Y |
| *nifX* | nitrogen fixation protein NifX | | **Y** | N | Y | N | Y | Y | N | Y | Y | Y | | N | Y | Y | Y | Y | Y |
| *nifN* | nitrogenase molybdenum-iron protein NifN | | **Y** | N | Y | N | Y | Y | N | Y | Y | Y | | N | Y | Y | Y | Y | Y |
| *nifE* | nitrogenase molybdenum-cofactor synthesis protein NifE | | **Y** | N | Y | N | Y | Y | N | Y | Y | Y | | N | Y | Y | Y | Y | Y |
| *nifY* | nitrogen fixation protein NifY | | **Y** | N | Y | N | Y | N | N | N | N | N | | N | Y | N | Y | Y | Y |
| *nifT* | nitrogen fixation protein NifT | | **Y** | N | Y | N | Y | Y | Y | Y | Y | Y | | N | Y | N | Y | Y | Y |
| *nifK* | nitrogenase molybdenum-iron protein beta chain | | **Y** | N | Y | N | Y | Y | Y | Y | Y | Y | | N | Y | N | Y | Y | Y |
| *nifD* | nitrogenase molybdenum-iron protein alpha chain | | **Y** | N | Y | N | Y | Y | Y | Y | Y | Y | | N | Y | N | Y | Y | Y |
| *nifH* | nitrogenase iron protein NifH | | **Y** | N | Y | N | Y | Y | Y | Y | Y | Y | | N | Y | N | Y | Y | Y |
| *nifA* | Nif-specific regulatory protein | | **Y** | N | Y | N | Y | Y | Y | Y | Y | N | | N | Y | Y | Y | Y | Y |
| *nifB* | nitrogen fixation protein NifB | | **Y** | N | Y | N | Y | Y | Y | Y | Y | N | | N | Y | Y | Y | Y | Y |
| *nifQ* | nitrogen fixation protein NifQ | | **Y** | N | Y | N | Y | Y | Y | Y | Y | N | | N | Y | Y | Y | Y | Y |
| Putative *nifR*-like | tRNA-dihydrouridine synthase, nitrogen regulation protein | | **Y** | Y | Y | N | Y | Y | Y | Y | Y | Y | | Y | Y | Y | Y | Y | Y |
| nifU | [Fe-S] cluster assembly protein NifU | | **Y** | Y | N | Y | Y | Y | Y | Y | Y | Y | | Y | Y | Y | Y | Y | Y |
| **Assimilatory nitrate reduction** | | | | | | | | | | | | | | | | | | | |
| *nasA* | assimilatory nitrate reductase catalytic subunit | | **Y** | Y | N | N | N | N | N | N | N | N | | N | N | N | N | N | N |
| *nirB* | assimilatory nitrite reductase NADH-dependent large subunit | | **Y** | Y | N | N | N | N | N | N | N | N | | N | N | N | N | N | N |
| *nirD* | assimilatory nitrite reductase NADH-dependent small subunit | | **Y** | Y | N | N | N | N | N | N | N | N | | N | N | N | N | N | N |
| **Dissimilatory nitrate reduction** | | | | | | | | | | | | | | | | | | | |
| *napC* | cytochrome c-type protein NapC | | **Y** | Y | Y | Y | Y | Y | Y | Y | Y | Y | | Y (X4) | Y | N | Y | Y | Y |
| *napB* | periplasmic nitrate reductase, electron transfer subunit | | **Y** | Y | N | N | Y | N | N | N | N | N | | Y | N | N | Y | Y | Y |
| *napH* | ferredoxin-type protein NapH | | **Y** | Y | N | Y | Y | N | N | N | N | Y | | Y (X2) | Y | Y | Y | Y | Y |
| *napG* | ferredoxin-type protein NapG | | **Y** | Y | N | Y | Y | N | N | N | N | Y | | Y (X2) | Y | Y | Y | Y | Y |
| *napA* | periplasmic nitrate reductase NapA | | **Y** | Y | N | Y | Y | N | N | N | N | Y | | Y | N | N | Y | Y | Y |
| *napD* | chaperone for the periplasmic nitrate reductase | | **Y** | N | N | N | N | N | N | N | N | N | | Y | N | N | Y | Y | Y |
| *napF* | ferredoxin-type protein NapF | | **N** | N | N | N | N | N | N | N | N | N | | Y | N | N | Y | Y | Y |
| putatie *narG* | putative respiratory nitrate reductase A, alpha chain | | **Y** | Y | Y | N | Y | Y | N | Y | Y | N | | N | Y | Y | N | N | N |
| putative *narH* | putative respiratory nitrate reductase A, beta chain | | **Y** | Y | Y | N | Y | Y | N | Y | Y | N | | N | Y | N | N | N | N |
| putative *narI* | putative respiratory nitrate reductase A, gamma chain | | **Y** | Y | Y | N | N | N | N | N | N | N | | N | N | N | N | N | Y |
| *nirS* | nitrite reductase (NO-forming) / hydroxylamine reductase | | **Y** | Y | Y | Y | Y | N | N | N | N | N | | N (?) | Y | Y | N | N | N |
| *nirN* | nitrite reductase associated c-type cytochorome | | **Y** | Y | Y | Y | Y | N | N | N | N | N | | Y | Y | Y | N | N | N |
| *nirC* | cytochrome c55X precursor | | **Y** | Y | Y | Y | Y | N | N | N | N | N | | Y | Y | Y | N | N | N |
| *nirF* | nitrite reductase heme d1 biosynthesis protein | | **Y** | Y | Y | Y | Y | N | N | N | N | N | | Y | Y | Y | N | N | N |
| *nirL* | heme d1 biosynthesis protein NirL | | **Y** | Y | Y | Y | Y | N | N | N | N | N | | Y | Y | Y | N | N | N |
| *nirG* | heme d1 biosynthesis protein NirG | | **Y** | Y | Y | Y | Y | N | N | N | N | N | | Y | Y | Y | N | N | N |
| *nirH* | nitrite reductase heme d1 biosynthesis protein | | **Y** | Y | Y | Y | Y | N | N | N | N | N | | Y | Y | Y | N | N | N |
| *nirJ* | heme d1 biosynthesis protein NirJ | | **Y** | Y | Y | Y | Y | N | N | N | N | N | | Y | Y | N | N | N | N |
| *norC* | nitric oxide reductase subunit C | | **N** | N | Y | N | N | N | Y | N | Y | Y | | N | Y | N | Y | Y | N |
| *norB* | nitric oxide reductase subunit B | | **N** | N | Y | N | N | N | Y | N | Y | Y | | N | Y | N | Y | Y | N |
| *norQ* | nitric oxide reductase NorQ protein | | **Y** | Y | Y | Y | N | N | Y | N | Y | Y | | N | Y | N | Y | Y | N |
| *norD* | nitric oxide reductase activation protein | | **Y** | Y | Y | Y | **N** | **N** | **Y** | **N** | **Y** | **Y** | | **N** | Y | **N** | Y | Y | N |
| *nosZ* | nitrous-oxide reductase | | **Y** | Y | Y | Y | Y | N | Y | N | N | N | | Y | Y | N | N | N | N |
| *nosD* | nitrous oxidase accessory protein | | **Y** | Y | Y | Y | Y | N | Y | N | N | N | | Y | Y | N | N | N | N |
| *nosF* | nitrous oxide reductase maturation protein | | **Y** | Y | Y | Y | Y | N | Y | N | N | N | | N | Y | N | N | N | N |
| *nosY* | probable ABC transporter permease protein NosY | | **Y** | Y | Y | Y | Y | N | Y | N | N | N | | Y | Y | N | N | N | N |
| *nosL* | copper-binding lipoprotein NosL | | **Y** | Y | Y | Y | Y | N | Y | N | N | N | | N | Y | N | N | N | N |
| **Sulfur** | | | | | | | | | | | | | | | | | | | |
| **Assimilatory sulfate reduction** | | | | | | | | | | | | | | | | | | | |
| *cysN* | sulfate adenylyltransferase, small subunit | | **N** | N | N | N | N | N | N | N | N | N | | N | N | N | Y | N | N |
| *cysD* | sulfate adenylyltransferase, large subunit | | **N** | N | N | N | N | N | N | N | N | N | | N | N | N | Y | N | N |
| *cysC* | APS kinase | | **Y** | Y | Y | Y | Y | Y | Y | Y | Y | Y | | Y | Y | Y | Y | Y | Y |
| *cysH* | PAPS reductase | | **N** | N | N | N | N | N | N | N | N | N | | N | N | Y | N | N | N |
| *cysI* | sulfite reductase | | **N** | N | N | N | N | N | N | N | N | N | | N | N | Y | N | N | N |
| **Cystein synthesis** | | | | | | | | | | | | | | | | | | | |
| *cysE* | serine O-acetyltransferase | | **Y** | Y | Y | Y | N | Y | Y | Y | Y | Y | | Y | Y | Y | Y | Y | Y |
| *cysK* | cysteine synthase A | | **Y** | Y | Y | Y | Y | Y | N | Y | Y | Y | | Y | Y | Y | Y | Y | Y |
| *cysM* | cysteine synthase B | | **Y** | Y | Y | Y | Y | Y | Y | Y | Y | Y | | Y | Y | Y | Y | Y | Y |
| **Dissimilatory sulfate/sulfite reduction and oxidation** | | | | | | | | | | | | | | | | | | | |
| *dsrA* | | dissimilatory sulfite reductase alpha subunit | **Y (x2)** | Y (x2) | Y | Y (x2) | Y | Y | N | Y | Y | Y | | Y (x2) | N | Y | Y | Y | Y |
| *dsrB* | | dissimilatory sulfite reductase beta subunit | **Y (x2)** | Y (x2) | Y | Y (x2) | Y | Y | N | Y | Y | Y | | Y (x2) | N | Y | Y | Y | Y |
| *dsrL* | | Iron-sulfur flavoprotein NADPH-dependent protein DsrL | **Y (x2)** | Y (x2) | Y | Y | Y | Y | N | Y | Y | Y | | Y (x2) | Y | Y | Y | Y | Y |
| *dsrC* | | dissimilatory sulfite reductase related protein | **Y** | Y (x2) | Y | Y | Y | Y (x2) | N | Y | Y | Y | | Y | Y | Y (x2) | Y | Y | Y |
| *dsrF* | | sulfur oxidation protein DsrF | **Y** | Y | Y | Y | Y | Y | N | Y | Y | Y | | Y | Y | Y | Y | Y | Y |
| *dsrE* | | sulfurtransferase DsrE | **Y** | Y | Y | Y (x2) | Y | Y | N | Y | Y | Y | | Y | Y | Y | Y | Y | Y |
| *dsrH* | | sulfur relay protein DsrH/TusB | **Y** | Y | Y | Y (x2) | Y | Y | N | Y | Y | Y | | Y | Y | Y | Y | Y | Y |
| *dsrJ* | | sulfur oxidizing c-type cytochrome DsrJ | **Y** | Y | Y | Y (x2) | Y | Y | N | Y | Y | Y | | Y | Y | Y | Y | Y | Y |
| *dsrO* | | sulfur oxidation protein DsrO | **Y** | Y | Y | Y (x2) | Y | Y | N | Y | Y | Y | | Y | Y | Y | Y | Y | Y |
| *dsrP* | | intracellular sulfur oxidation protein DsrP | **Y** | Y | Y | Y (x2) | Y | Y | N | Y | Y | Y | | Y | Y | Y | Y | Y | Y |
| *aprA* | | adenylylsulfate reductase, subunit A | **Y** | Y | Y | Y | Y | N | Y | Y | N | N | | Y | Y | Y | N | N | Y |
| *aprB* | | adenylylsulfate reductase, subunit B | **Y** | Y | Y | Y | Y | N | Y | Y | Y | Y | | Y | Y | Y | N | N | Y (x2) |
| *sat* | | sulfate adenylyltransferase, disimilatory type | **Y** | Y | Y | Y | Y | Y | Y | Y | Y | Y | | Y | Y | Y | N | Y | Y |
| **SOX system** | | | | | | | | | | | | | | | | | | | |
| *soxB* | | sulfate thiol esterase | **N** | N | N | N | N | N | N | N | N | N | | N | N | Y | Y | Y | Y |
| *soxA* | | L-cysteine S-thiosulfotransferase | **N** | N | N | N | N | N | N | N | N | N | | N | N | Y | Y (x2) | Y | Y |
| *soxZ* | | sulfur-oxidizing protein SoxZ | **Y** | Y | Y | Y | Y | Y | Y | Y | Y | N | | Y (x2) | Y | Y (x2) | Y (x3) | Y (x2) | Y (x2) |
| *soxY* | | sulfur-oxidizing protein SoxY | **Y** | Y | Y | Y | Y | Y | Y | Y | Y | Y | | Y (x2) | Y | Y (x2) | Y (x3) | Y (x3) | Y (x2) |
| *soxX* | | L-cysteine S-thiosulfotransferase | **N** | N | N | N | N | N | N | N | N | N | | N | N | Y | Y (x2) | Y (x2) | Y |
| *soxC* | | sulfane dehydrogenase subunit SoxC | **N** | N | N | N | N | N | N | N | N | N | | N | N | N | N | N | N |
| *soxD* | | S-disulfanyl-L-cysteine oxidoreductase SoxD | **N** | N | N | N | N | N | N | N | N | N | | N | N | N | N | N | N |
| - | | Thioredoxin-related protein | **N** | N | N | N | N | N | N | N | N | N | | N | N | N | Y | Y | N |
| *soxW* | | Thioredoxin-like protein | **N** | N | N | N | N | N | N | N | N | N | | N | N | Y | Y | Y | Y |
| **Hdr-like or QmoABC-like enzymes, which may be involved in sulfide oxidation of sulfate reduction** | | | | | | | | | | | | | | | | | | | |
| *hdrA1*/  *qmoA* | | putative heterodisulfide reductase subunit A | **Y** | Y | Y | Y | Y | Y | N | Y | Y | Y | | N | Y | N | N | N | N |
| *hdrA2/*  *qmoB* | | putative heterodisulfide reductase subunit A | **Y** | Y | Y | Y | Y | Y | N | Y | Y | Y | | N | Y | N | N | N | N |
| *hdrB* | | putative heterodisulfide reductase subunit B | **Y** | Y | Y | Y | Y | Y | N | Y | Y | Y | | N | Y | N | N | N | N |
| *hdrC* | | putative heterodisulfide reductase subunit C | **Y** | Y | Y | Y | Y | Y | N | Y | Y | Y | | N | Y | N | N | N | N |
| **Sulfide oxidation** | | | | | | | | | | | | | | | | | | | |
| *fccA* | sulfide dehydrogenase (flavocytochrome), cytochrome c subunit | | **Y** | Y | Y | N | Y | Y | Y | Y | Y | Y | | Y | Y | Y | Y | Y | Y |
| *fccB* | sulfide dehydrogenase (flavocytochrome), flavoprotein subunit | | **Y** | Y | Y | N | Y | Y | Y | Y | Y | Y | | Y | Y | Y | Y | Y | Y |
| **Phosphorus** | | | | | | | | | | | | | | | | | | | |
| **Phosphate uptake** | | | | | | | | | | | | | | | | | | | |
| *pstD* | phosphate ABC transporter permease subunit | | **Y (x2)** | Y (x2) | Y (x2) | Y | Y | Y | Y (x2) | Y | Y | Y | | N | Y | Y | Y | Y | Y |
| *pstA* | phosphate ABC transporter permease PstA | | **Y (x2)** | Y (x2) | Y (x2) | Y | Y | Y | Y (x2) | Y | Y | Y | | N | Y | Y | Y | Y | Y |
| *pstB* | phosphate ABC transporter ATP-binding protein | | **Y (x4)** | Y (x4) | Y (x2) | Y (x2) | Y (x2) | Y | Y (x2) | Y | Y | Y | | N | Y | Y | Y | Y | Y |
| *phoU* | phosphate uptake regulator | | **Y (x2)** | Y (x2) | Y (x2) | Y | Y | Y | Y (x2) | Y | Y | Y (x2) | | N | Y | Y (x2) | Y | Y | Y (x2) |
| *-* | rhodanese-like domain-containing protein of unkn. function | | **Y** | Y | Y | Y | Y | Y | N | Y | N | N | | Y | N | N | Y | Y | Y (x2) |
| *pstS* | phosphate ABC transporter substrate-binding protein | | **Y** | Y (x2) | Y (x4) | Y | Y (x2) | Y (x2) | Y (x2) | Y (x2) | N | N | | Y | Y (x2) | Y | Y | Y | Y |
| *phoR* | OmpR family, sensor histidine kinase | | **Y (x3)** | Y (x3) | Y (x3) | Y (x2) | Y | N | N | N | N | N | | Y (x2) | N | N | Y | Y | Y |
| *phoB* | OmpR family, response regulator | | **Y (x3)** | Y (x4) | Y (x4) | Y (x2) | Y (x3) | Y | N | Y (x3) | Y (x2) | N | | Y (x2) | N | N | Y | Y | Y |
| **Phosphonate/phosphite uptake** | | | | | | | | | | | | | | | | | | | |
| *phnD1* | phosphonate-binding periplasmic protein | | **N** | N | N | N | N | N | N | N | N | N | | Y | N | N | Y | Y | Y |
| *phnA* | alkylphosphonate utilization protein | | **N** | N | N | N | N | N | N | N | N | N | | Y | N | N | Y | Y | Y |
| *phnD2* | phosphonate-binding periplasmic protein | | **Y** | N | N | N | N | Y | N | N | N | N | | N | N | N | Y | Y | Y |
| *phnD3* | putative periplasmic phosphonate-binding protein | | **N** | N | N | N | N | N | N | N | N | N | | N | N | N | Y | Y | Y |
| *phnD4* | putative periplasmic phosphonate-binding protein | | **N** | N | N | N | N | N | N | N | N | N | | N | N | N | Y | Y | Y |
| *phnD5* | putative periplasmic phosphonate-binding protein | | **N** | **N** | **N** | **N** | **N** | **N** | **N** | **N** | **N** | **N** | | **N** | **N** | **N** | Y | Y | Y |
| *phnD6* | putative periplasmic phosphonate-binding protein | | **Y** | **Y** | N | **N** | N | N | N | N | N | **N** | | N | N | Y | Y | Y | Y |
| **Polyphosphate synthesis and hydrolysis** | | | | | | | | | | | | | | | | | | | |
| *ppk1* | polyposphate kinase 1 | | **N** | **N** | **N** | **N** | **N** | **N** | **N** | **N** | **N** | **N** | | **N** | **N** | **N** | N | N | **N** |
| *ppk2* | polyposphate kinase 2 | | **Y (x2)** | Y (x3) | Y (x2) | Y (x3) | Y (x4) | Y (x3) | Y (x2) | Y (x3) | Y (x3) | Y (x3) | | Y | Y (x3) | N | Y (x2) | Y (x2) | Y (x3) |
| *pap* | Poly P:AMP Phosphotransferase | | **N** | **N** | **N** | **N** | **N** | **N** | **N** | **N** | **N** | **N** | | **N** | **N** | **N** | N | N | Y |
| *ppx* | exopolyphosphatase | | **N** | **N** | **N** | **N** | **N** | **Y** | **Y** | **Y** | **Y** | **Y** | | **N** | **Y** | **N** | Y | Y | Y |
| **Oxygen stress response** | | | | | | | | | | | | | | | | | | | |
| *sodA/B* | superoxide dismutase [Fe/Mn] | | **Y** | Y | Y | Y | Y | N | N | N | N | N | | N | N | Y | N | Y | Y |
| *katG* | catalase-peroxidase I | | **Y** | Y | N | N | N | N | N | N | N | N | | N | N | N | N | N | N |
| *katE* | catalase II | | **N** | N | Y | N | N | Y | Y | Y | N | N | | N | N | N | N | N | Y |
| *ccp1* | cytochrome c peroxidase | | **Y** | Y | Y | N | N | N | N | N | N | N | | N | Y | N | Y | Y | Y |
| *ccp2* | cytochrome c peroxidase | | **Y** | N | N | N | N | N | N | N | N | N | | N | Y | N | Y | Y | Y |
| *ccp3* | cytochrome c peroxidase | | **N** | N | N | N | N | N | N | N | N | N | | N | N | N | Y | Y | Y |
| *bcp* | thiol peroxidase | | **Y** | Y | Y | Y | N | Y | Y | Y | Y | Y | | Y | Y | Y | Y | Y | Y |
| *ahpC-like* | putative alkyl hydroperoxide reductase C subunit | | **Y** | Y | Y | Y | Y | Y | Y | Y | Y | Y | | Y | N | Y | Y | Y | Y |
| **Transport** | | | | | | | | | | | | | | | | | | | |
| **ABC transport systems** | | | | | | | | | | | | | | | | | | | |
| *modA* | molybdate transport system substrate-binding protein | | **Y** | Y | Y | Y | Y | Y | Y | Y | Y | Y | | Y | Y | Y | Y | Y | Y |
| *modB* | molybdate transport system permease protein | | **Y** | Y | Y | Y | Y | Y | Y | Y | Y | Y | | Y | Y | Y | Y | Y | Y |
| *modC* | molybdate transport system ATP-binding protein | | **Y** | Y | N | Y | Y | Y | Y | Y | Y | Y | | Y | Y | Y | Y | Y | Y |
| *znuB* | zinc ABC transporter, inner membrane permease protein | | **Y** | Y | Y(x2) | Y | Y | Y | Y | Y | Y | Y | | Y | Y | Y | Y | Y | Y |
| *znuC* | zink transport system ATP-binding protein | | **Y** | Y | Y | Y | Y | Y | Y | Y | Y | Y | | Y | Y | Y | Y | Y | Y |
| *znuA* | zinc transport system substrate-binding protein | | **Y** | Y | Y | Y | Y (x2) | Y | Y | Y | Y | Y | | Y | Y | Y | Y | Y | Y |
| *cbiO* | cobalt ABC transporter ATP-binding protein | | **Y** | Y | Y | N | N | Y | Y | Y | Y | Y | | N | Y | Y | Y | Y | Y |
| *cbiQ* | cobalt ABC transporter, inner membrane subunit | | **N** | N | Y | N | N | Y | Y | Y | Y | Y | | N | Y | Y | Y | N | N |
| *cbiM* | cobalt/nickel transport system permease protein | | **N** | N | Y | N | N | Y | Y | Y | Y | Y | | N | Y | Y | Y | N | N |
| *cbiN* | cobalt/nickel transport protein | | **N** | N | N | N | N | Y | Y | Y | Y | Y | | N | Y | N | Y | N | N |
| *livK* | branched-chain amino acid transport system substrate-binding protein | | **Y** | Y | Y | Y | Y | Y | N | Y | Y | Y | | Y | Y | Y | Y | Y | Y |
| *livH* | branched-chain amino acid ABC transporter permease | | **Y** | Y | Y | Y | Y | Y | N | Y | Y | Y | | Y | Y | Y | Y | Y | Y |
| *livM* | branched-chain amino acid transport system permease | | **Y** | Y | Y | Y | Y | Y | N | Y | Y | Y | | Y | Y | Y | Y | Y | Y |
| *livG* | branched-chain amino acid transport system ATP-binding protein | | **Y** | Y | Y | Y | Y | Y | N | Y | Y | Y | | Y | Y | Y | Y | Y | Y |
| *livF* | branched-chain amino acid transport system ATP-binding protein | | **Y** | Y | Y | Y | Y | Y | N | Y | Y | Y | | Y | Y | Y | Y | Y | Y |
| *urtA* | urea transport system substrate-binding protein | | **Y** | N | Y | Y | N | N | N | N | N | N | | N | Y | N | Y | N | N |
| *urtB* | urea transport system substrate-binding protein | | **Y** | N | Y | N | N | N | N | N | N | N | | N | Y | N | Y | N | N |
| *urtC* | urea transport system permease protein | | **Y** | N | Y | N | N | N | N | N | N | N | | N | Y | N | Y | N | N |
| *urtD* | urea transport system ATP-binding protein | | **Y** | N | Y | N | N | N | N | N | N | N | | N | Y | N | Y | N | N |
| *urtE* | urea transport system ATP-binding protein | | **Y** | N | Y | N | N | N | N | N | N | N | | N | Y | N | Y | N | N |
| *gltI* | glutamate aspartate periplasmic binding protein precursor | | **Y** | Y | Y | N | Y | N | N | N | N | N | | N | N | N | N | N | N |
| *gltK* | glutamate aspartate transport system permease protein | | **Y** | Y | Y | N | Y | N | N | N | N | N | | N | N | N | N | N | N |
| *gltL* | glutamate aspartate transport ATP-binding protein | | **Y** | Y | Y | N | Y | N | N | N | N | N | | N | N | N | N | N | N |
| *nrtA* | nitrate/nitrite transport system substrate-binding protein | | **Y** | Y | N | N | N | N | N | N | N | N | | N | N | N | N | N | N |
| *nrtB* | nitrate/nitrite transport system permease protein | | **Y** | Y | N | N | N | N | N | N | N | N | | N | N | N | N | N | N |
| *nrtC* | nitrate/nitrite transport system ATP-binding protein | | **Y** | Y | N | N | N | N | N | N | N | N | | N | N | N | N | N | N |
| *afuA* | iron(III) transport system substrate-binding protein | | **Y** | Y | N | N | N | N | N | N | N | N | | N | N | N | N | N | Y |
| *afuB* | iron(III) transport system permease protein | | **Y** | Y | N | N | N | N | N | N | N | N | | N | N | N | N | N | Y |
| *afuC* | iron(III) transport system ATP-binding protein | | **Y** | Y | N | N | N | N | N | N | N | N | | N | N | N | N | N | Y |
| **Other transporters** | | | | | | | | | | | | | | | | | | | |
| *mgtE* | Mg/Co/Ni transporter | | **Y (x3)** | Y (x3) | Y (x3) | Y (x3) | Y (x3) | N | Y | N | Y | Y | | N | N | Y | Y | N | Y |
| *corA/zntB*-like | Mg/Co/Ni transporter or zinc transporter ZntB | | **Y** | Y | Y | Y | Y (x2) | N | Y | N | N | N | | Y | N | N | Y | N | Y |
| *cbtC* | predicted Co transporter | | **N** | Y | N | Y | Y | N | N | N | N | N | | Y | N | N | N | N | N |
| *actP* | cation/acetate symporter | | **Y** | Y | Y | Y | Y | Y | N | Y | Y | Y | | Y | N | Y | Y | Y | Y |
| *sulP1* | putative sulfate permease, SulP family | | **Y** | Y | Y | Y | Y | Y | Y | Y | Y | Y | | Y | Y | Y | Y | Y | Y |
| *sulP2* | putative sulfate permease, SulP family | | **Y** | Y | Y | Y | Y | Y | N | Y | Y | Y | | N | Y | Y | Y | Y | Y |
| *sulP3* | putative sulfate permease, SulP family | | **Y** | Y | Y | Y | Y | Y | N | Y | Y | Y | | N | Y | Y | Y | N | Y |
| *sulP4* | putative sulfate permease, SulP family | | **Y** | N | Y | N | Y | Y | N | Y | Y | Y | | N | N | N | Y | N | N |
| *pitA/B*-like | low-affinity metal phosphate:H(+) symporter PitB | | **Y (x5)** | Y (x5) | Y (x5) | Y (x5) | Y (x5) | N | N | N | N | N | | Y (x2) | N | N | Y | Y | Y |
| **Ferrous iron transporters** | | | | | | | | | | | | | | | | | | | |
| *feoA1* | ferrous iron transport protein | | **N** | Y | Y | **N** | Y | Y | Y | Y | **N** | **N** | | **N** | Y | **N** | Y | N | Y |
| *feoB1* | ferrous iron transport protein | | **Y** | Y | Y | **N** | Y | Y | Y | Y | **N** | **N** | | Y | Y | **N** | N | N | Y |
| *feoA2* | ferrous iron transport protein | | **Y** | Y | Y | **N** | Y | Y | Y | Y | **N** | **N** | | Y | Y | **N** | Y | Y | Y |
| *feoB2* | ferrous iron transport protein | | **Y** | Y | Y | Y | Y | Y | Y | Y | Y | Y | | Y | Y | Y | Y | Y | Y |
